# Physical exercise in combination with audiovisual stimulation alleviates cognitive and affective impairments in Alzheimer’s disease model mice via restoring lysosomal membrane integrity

**DOI:** 10.1186/s13195-026-02075-8

**Published:** 2026-05-29

**Authors:** Jun Jia, Guomin Xie, Wu Zheng, Chunshuang Xu, Yuxin Xia, Qiaoxia Hu, Binbin Xiang, Xinkai Zhou, Anqi Chen, Xiaoping Chen, Qinwen Wang, Yingsong Zhou, Shujun Xu

**Affiliations:** 1 Department of Neurology, The Affiliated Lihuili Hospital of Ningbo University, Ningbo, Zhejiang 315040 China; 2 Department of Physiology and Pathophysiology, Health Science Center, Ningbo University, Ningbo, Zhejiang 315211 China; 3 Faculty of Sports Science, Ningbo University, Ningbo, Zhejiang 315211 China; 4 Department of Anesthesiology, Women and Children’s Hospital, Ningbo University, Ningbo, Zhejiang 315012 China; 5 Department of Geriatrics, The First Affiliated Hospital of Ningbo University, Ningbo, Zhejiang 315010 China; 6 China Institute of Sport Science, Beijing, 100061 China

**Keywords:** Alzheimer's disease, Exercise, Audiovisual stimulation, Autophagy, Lysosome, Neuroinflammation

## Abstract

**Background:**

Alzheimer’s disease (AD) is a progressive disorder characterized by cognitive decline. Physical exercise and audiovisual stimulation have gained increasing concern for their potential to mitigate AD pathology. However, the therapeutic advantages of combining these interventions and the precise molecular mechanisms underlying these strategies need further demonstration.

**Objectives:**

This study aimed to assess the protective effects and underlying mechanisms of physical exercise combined with audiovisual stimulation on cognitive and affective functions, as well as on pathological alterations in AD mice.

**Methods:**

Both AD model mice established by injecting Aβ₄₂ oligomers into hippocampus and APP/PS1 AD transgenic mice were used. Mice were subjected to treadmill training, 40 Hz audio-visual stimulation, or a combination of these interventions, respectively. After the interventions, the cognitive and anxiety/depression-like behaviors were evaluated by novel object recognition, morris water maze, open field, tail suspension, or forced swimming, respectively. Quantitative proteomics combined with molecular analyses and transmission electron microscopy were used to systematically evaluate the underlying mechanism of multimodal interventions in AD model mice.

**Results:**

The multimodal intervention significantly prevented cognitive impairment and ameliorated anxiety/depression-like behaviors of APP/PS1 AD transgenic mice and AD model mice induced by injecting Aβ₄₂ oligomers, outperforming single-modality treatments. It markedly diminished hippocampal accumulation of β-amyloid (Aβ) and tau phosphorylation in AD mice. Multiple interventions also reversed synapse loss of AD mice. Proteomic analyses revealed that multimodal intervention exerted a more comprehensive restoration of dysregulated proteins in AD mice compared to single-modality interventions. The interventions have synergetic effects in decreasing inflammation reactions and restoring the autophagy-lysosomal function. Multimodal intervention upregulated the expression TFEB, and concurrently increased HSPA1L expression to restore lysosomal membrane integrity. The degradation function of lysosomes was also improved by multimodal intervention as revealed by the decreased LC3II/I ratio, reduced p62 level, as well as alleviated lysosome enlargement in AD mice. Upregulation of HSPA1L reversed the disruption of lysosome membrane integrity of AD transegenic mice, thereby reversed the increased accumulation of Aβ and cognitive defects of AD.

**Conclusion:**

Physical exercise and audiovisual stimulation exert synergistic effects in decreasing the inflammation reaction and maintaining autophagy-lysosomal homeostasis by increasing the biogenesis of lysosomes and restoring the integrity of lysosome membrane, thereby reducing Aβ deposition and cognitive defect of AD mice. This study highlights the significant therapeutic potential of multimodal, non-pharmacological strategies for Alzheimer’s disease.

**Supplementary Information:**

The online version contains supplementary material available at 10.1186/s13195-026-02075-8.

## Introduction

Alzheimer’s disease (AD) is a common neurodegenerative disorder characterized by progressive memory loss, cognitive decline, and visuospatial impairments. With rapid global population aging, the prevalence and socioeconomic burden of AD continue to rise sharply, projected to affect over 130 million individuals worldwide by 2050 [1–3]. Pathological hallmarks of AD include β-amyloid (Aβ) deposition, hyperphosphorylated tau protein, synaptic dysfunction, and neuroinflammation [4, 5]. Although currently available pharmacological treatments can alleviate some cognitive and functional symptoms, their effects on modifying the underlying disease progression remain limited, and there remains an urgent need for more effective or low-cost interventions [6, 7].

It has been demonstrated that non-pharmacological therapies, particularly physical exercise and audiovisual stimulation, have significant potential in ameliorating AD symptoms [8, 9]. Exercise enhanced brain plasticity, effectively reduced Aβ deposition and inflammation, thereby improving cognitive outcomes in AD model mice [10–12]. Additionally, recent studies suggest that 40 Hz audiovisual stimulation may confer therapeutic benefits by inducing gamma oscillations, facilitating Aβ clearance, and alleviating cognitive deficits [13–16]. Given the promising results of these individual interventions, there is growing interest in exploring combined strategies that target multiple factors simultaneously [17, 18]. However, the synergistic effects and precise molecular mechanisms underlying the therapeutic benefits of multimodal interventions in AD remain incompletely understood.

Dysfunction of the autophagy-lysosomal system has emerged as a key factor in AD pathogenesis, contributing to impaired clearance of neurotoxic proteins such as Aβ and hyperphosphorylated Tau [19, 20]. In addition to causing defects in protein degradation, impairment of the autophagy-lysosome pathway activated inflammatory signaling pathways and exacerbated neuronal damage. Recent studies have demonstrated that enhancing autophagy activity can reduce neuroinflammation, decrease the accumulation of pathological proteins, and improve cognitive function in AD mice [21–23]. Therapeutic strategies aimed at restoring autophagy-lysosomal function have shown promise in reducing protein aggregates and mitigating neuronal damage [24–26]. However, whether physical exercise and audiovisual stimulation have synergistic protective effects in reversing autophagy-lysosomal system disturbance in AD mice and the precise molecular mechanisms remain unclear.

In the present study, multiple behavioral assessments, quantitative proteomics, molecular analyses and transmission electron microscopy were combined to systematically evaluate the protective effects and underlying mechanism of multimodal interventions in AD model mice.

## Materials and methods

### Animal

**Fig. 1:**
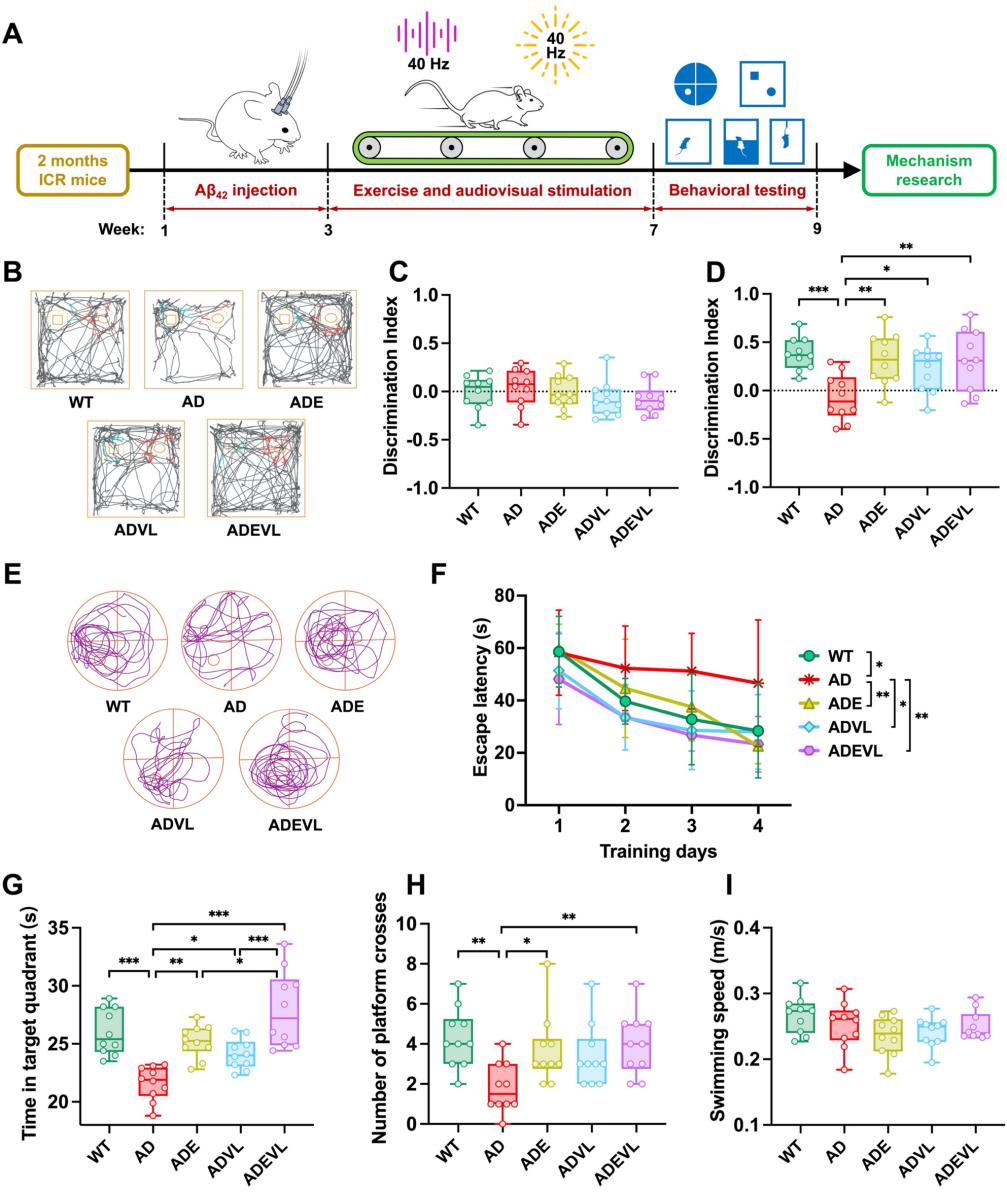
Multimodal intervention reversed the cognitive impairments of AD mice. **A** Schematic illustration of the experimental timeline. **B** Representative trajectories during the retention session in the novel object recognition (NOR). **C** Discrimination index during the training session in the NOR. **D** Discrimination index during the retention session in the NOR.** E** Representative swimming trajectories during the spatial probe test in the Morris water maze (MWM). **F **Escape latency during spatial acquisition training in the MWM. **G** Time spent in the target quadrant during the spatial probe test in the MWM. **H** Number of target platform crosses during the spatial probe test in the MWM. **I** Swimming speed during the spatial probe test in the MWM. *n* = 10 per group, *P < 0. 05, **P < 0. 01, ***P < 0. 001 (one-way ANOVA followed by Tukey’s post hoc test or two-way ANOVA followed by Dunnett’s post hoc test)

Seven-month-old male APP/PS1 transgenic mice were purchased from Aniphe Biolab, two-month-old male ICR mice were purchased from Vital River Laboratory Animal Technology Co., Ltd. (Jiaxing, China), and housed at the Animal Experiment Center of Ningbo University (12-h light/dark cycle, 22 ± 2 °C, 50 ± 10% humidity) with ad libitum access to food and water. All procedures were approved by the Institutional Animal Care and Use Committee of Ningbo University (NBU20230158) and followed NIH guidelines. Mice were randomly divided into five groups: wild-type control (WT), AD model (AD), AD with exercise (ADE), AD with 40 Hz voice and light (ADVL), and AD with multimodal intervention (ADEVL). Under isoflurane anesthesia, mice were placed in a stereotaxic frame, and bilateral cannulas were implanted into the hippocampus (AP − 1. 7 mm, ML ± 1. 0 mm, DV − 1. 5 mm from the pia). After 7 days of recovery, AD model mice received intrahippocampal injections of Aβ_42_ (4 µmol/kg) once daily for 3 days, while WT mice received vehicle. Following another 7-day recovery, mice underwent a 4-week intervention: the ADE group received treadmill training (12 m/min, 46 min/day, 6 days/week), the ADVL group received 40 Hz sound and light stimulation (1 h/day, 6 days/week), and the ADEVL group received both interventions simultaneously. Interventions were administrated at night cycle during 20:00 and 21:00 to avoid disruption of the mice sleeping behavior. Behavior tests were administrated during day time. Molecular analyses were performed after the behavioral testing was completed (Fig. 1A).

### Aβ42 oligomer preparation

Soluble Aβ_42_ oligomers were prepared as previously described [27]. 1 mg of Aβ_1−42_ (Bachem, Cat # H-1368. 1000) was dissolved in 400 µl ice-cold HFIP (Aladdin, Cat # K1625063) and incubated at room temperature for 20 min. A 100 µl aliquot was diluted in 900 µl deionized water (final concentration: 25 g/L), centrifuged at 14, 000 g for 15 min, and the supernatant was collected. After complete HFIP evaporation, the solution was stirred at room temperature for 48 h to yield a 50 µM Aβ_42_ solution, which was stored at 4 ℃.

### Behavioral tests

#### Novel object recognition

The novel object recognition (NOR) task was conducted in an open-field arena (40 × 40 × 40 cm) over two days following non-pharmacological interventions and consisted of a familiarization section and a test section. On the first day (familiarization), each mouse was allowed to explore two identical objects for 5 min. On the second day (test), one familiar object was replaced with a novel object that differed in both shape and material, and the mouse was allowed to explore freely for 5 min. To eliminate olfactory cues, the arena and objects were thoroughly cleaned with 70% ethanol between trials. Exploration was defined as sniffing or touching an object while the mouse’s nose was within 2 cm of it. To evaluate recognition memory, the discrimination index was calculated as (Tnovel-Tfamiliar) / (Tnovel+Tfamiliar). Tnovel and Tfamiliar represent the time spent exploring the novel and familiar objects, respectively, with a higher discrimination index reflecting superior cognitive performance.

#### Morris water maze

The Morris water maze (MWM) test was conducted as previously described [28]. Briefly, the apparatus consisted of a circular pool (diameter = 120 cm) filled with opaque water maintained at 22 ± 1 °C. Spatial learning was assessed during the place‑navigation section by recording the latency to locate a hidden platform positioned ~ 1 cm below the water surface. From day 1 to day 4, each mouse completed four trials per day (intertrial interval, 20–30 min) and was allowed a maximum of 90 s per trial to find the platform. On day 5, the platform was removed, and a probe trial was conducted. Behavioral metrics, including escape latency during training (time from water entry to climbing onto the platform), time spent in the target quadrant, number of crossings over the former platform location, and average swimming speed, were extracted and analyzed by the ANY‑maze behavior analysis system. All assessments were performed by an investigator blinded to the experimental groups.

#### Open field

Each mouse was carefully placed in the center of a square open‑field arena (40 × 40 × 40 cm) and allowed to explore freely for 5 min, with locomotor activity recorded by an overhead video camera. After each trial, feces were removed, and the arena was wiped with 75% ethanol to eliminate residual odors. The next mouse was tested only after the enclosure had completely dried. Exploration trajectories and the duration of time spent in the central zone were quantified using the ANY-maze behavioral tracking system.

#### Tail suspension

For the tail suspension test, adhesive tape was applied approximately 1 cm from the tip of the tail and attached to the top of the suspension apparatus, ensuring the mouse’s head remained 10–15 cm above the ground. Each mouse was suspended for 6 min, with activity recorded via video camera. The apparatus was cleaned with 75% ethanol and allowed to dry between tests. Immobility time during the final 4 min was analyzed using the ANY-maze system, with immobility defined as a complete absence.

#### Forced swimming test

Each mouse was placed in a transparent plastic cylinder (25 cm in height, 18 cm in diameter) filled with 15 cm of water maintained at 22 ± 1 °C for 6 min, with behavior recorded via video camera. The water was replaced after each trial to eliminate odor cues. Immobility time during the final 4 min was analyzed using the ANY-maze system. Immobility was defined as floating with the head above water or making only minimal movements to keep the head afloat, such as slight paddling with one hind limb.

#### Proteomic analysis

Hippocampal tissues were rapidly frozen in liquid nitrogen and lysed using RIPA buffer. After ultrasonic disruption and centrifugation, the supernatant was collected, and protein concentration was determined using the BCA assay. Equal amounts of protein were then enriched for low-abundance proteins using magnetic beads, followed by enzymatic digestion. Peptides were desalted using C18 cartridges, resuspended, and quantified by measuring the optical density at 280 nm. Subsequently, the samples were spiked with iRT standard peptides. Protein samples were fractionated by HPLC and separated on a Vanquish Neo system using a C18 column (Thermo Scientific, ES906, 1. 9 μm, 150 μm × 15 cm), applying a 0. 1% formic acid acetonitrile gradient (up to 80%). Peptides were analyzed by nanoLC-MS/MS in DIA mode on a Thermo Astral mass spectrometer. MS1 scans were acquired over the range of 380–980 m/z at a resolution of 240, 000 (at m/z 200), with a normalized AGC target set at 500% and a maximum injection time of 5 ms. MS2 spectra were acquired using 2 m/z isolation windows, HCD collision energy of 25 eV, an AGC target of 500%, and an injection time of 3 ms. Data processing was performed using DIA-NN, with fixed carbamidomethyl (C) modifications and variable modifications including oxidation (M) and N-terminal acetylation. A false discovery rate (FDR) was controlled below 1%.

Differentially expressed proteins (DEPs) were identified using quantitative proteomic data. Proteins with absolute log2 fold change (|log2FC|) > 0.6 and *P-*value < 0.05 were identified as significantly differentially expressed. Volcano plots were constructed to visualize the overall distribution of DEPs. The x-axis represented log2 fold change, and the y-axis represented −log10 *P-*value. Up-regulated, down-regulated, and non-significantly changed proteins were distinguished by different colors. Hierarchical clustering was performed to visualize the expression patterns of DEPs across groups. Normalized expression levels were used for cluster analysis. The color gradient indicated relative protein abundance, with red representing high expression and blue representing low expression. Subsequently, Gene Ontology (GO) annotation, Kyoto Encyclopedia of Genes and Genomes (KEGG) pathway enrichment, and protein-protein interaction (PPI) network analysis were performed to identify biological functions and critical signaling pathways. Gene Set Enrichment Analysis (GSEA) was performed to analyze the biological functions of proteins based on their overall expression profiles. The Gene Ontology (GO) gene sets were selected as reference gene sets. Statistical significance was assessed by a permutation test. Terms with normalized enrichment score (NES) > 1 or < − 1 and *P*-value < 0.05 were considered significantly enriched. All data analysis was conducted using R version 4.4.3.

### Western blot

Hippocampal tissues were homogenized in high-efficiency RIPA lysis buffer supplemented with 1% protease and phosphatase inhibitor cocktails. The lysates were sonicated, incubated on ice for 30 min, and then centrifuged at 12, 000 rpm for 30 min at 4 °C. The supernatant was collected, and protein concentrations were determined using a BCA protein assay kit. Following quantification, one-quarter volume of 5× SDS-PAGE loading buffer was added to each sample. Proteins (40 µg per lane) were separated by SDS-PAGE using the gel preparation and transferred onto 0.45/0.2 μm PVDF membranes with rapid transfer buffer. Membranes were blocked with ready-to-use blocking buffer at room temperature for 5 min, then incubated overnight at 4 °C with [Phospho- microtubule associated protein tau (p-Tau Ser396, 1:1000, Abcam, Cat # ab32057), Microtubule associated protein tau (Tau, 1:1000, Cell Signaling Technology, Cat # 46687), Lysosomal Associated Membrane Protein 1 (LAMP1, 1:1000, Cell Signaling Technology, Cat # 3243), Transcription Factor EB (TFEB, 1:1000, Proteintech, Cat # 13372-1-AP), Microtubule Associated Protein 1 Light Chain 3 Beta (LC3B, 1:1000, Novus, Cat # NB600-1384), Sequestosome 1 (p62/SQSTM1, 1:1000, Cell Signaling Technology, Cat # 5114), Heat Shock Protein A 1 Like (HSPA1L, 1:1000, Proteintech, Cat # 66780-1-Ig) and Glyceraldehyde-3-Phosphate Dehydrogenase (GAPDH, 1:50000, Proteintech, Cat # 60004-1-Ig) ], respectively. After primary antibody incubation and washing, membranes were incubated with HR*P-*conjugated secondary antibodies [anti-rabbit IgG (1:3000, Cell Signaling Technology, Cat # 7074), anti-mouse IgG (1:3000, Cell Signaling Technology, Cat # 7076) ] for 1 h at room temperature, respectively. Protein bands were visualized using Western Bright ECL and captured with a chemiluminescence imaging system.

### Immunohistochemistry and immunocytochemistry

For brain tissue, mice were anesthetized and transcardially perfused with PBS followed by 30 mL of 4% paraformaldehyde (PFA). Brains were collected, post-fixed overnight at 4 °C, cryoprotected in 15% and 30% sucrose until fully submerged, embedded in OCT, frozen, and sectioned at 25–30 μm using the Microtome Cryostat (Leica, CM1950). Sections were stored at -20 °C in preservation solution. Brain sections were washed with PBS, subjected to antigen retrieval in a 95 °C water bath for 20 min, cooled to room temperature, and blocked for 1 h. For cell samples, cells on coverslips were fixed with 4% PFA at room temperature for 20 min and blocked similarly. All samples were then incubated overnight at 4 °C with primary antibodies [Amyloid-beta Peptide 1–42 (Aβ 1–42, 1:500, Abcam, Cat # ab201061); Galectin-3 (Gal3, 1:500, Proteintech, Cat # 27874-1-AP); Heat Shock Protein A 1 Like (HSPA1L, 1:500, Proteintech, Cat # 66780-1-Ig); Synaptophysin (SYP, 1:500, Proteintech, Cat # 67864-1-Ig); Postsynaptic Density Protein 95 (PSD95, 1:500, Cell Signaling Technology, Cat # 3450); Glial Fibrillary Acidic Protein (GFAP, 1:500, Cell Signaling Technology, Cat # 3670); Complement Component 1q (C1q, 1:500, Abcam, Cat # ab182451); Ionized Calcium Binding Adaptor Molecule 1 (Iba1, 1:300, Abcam, Cat # ab283319); Neuronal Nuclear Protein (NeuN, 1:300, Abcam, Cat # ab104224); Transmembrane Protein 119 (TMEM119, 1:500, Abcam, Cat # ab209064); Lysosomal Associated Membrane Protein 1 (LAMP1, 1:500, Abcam, Cat # ab208943)]. After primary antibody incubation and washing with PBS, samples were incubated for 1 h at room temperature with secondary antibodies [Alexa Fluor 488-conjugated goat anti-rabbit IgG H&L (1:500, Abcam, Cat # ab150077), Alexa Fluor 488-conjugated goat anti-mouse IgG (1:500, Beyotime, Cat # A0428), or Alexa Fluor 647-conjugated goat anti-rabbit IgG (1:500, Beyotime, Cat # A0468) ]. Finally, samples were mounted using DAPI-containing anti-fluorescence quencher, sealed, and imaged using a confocal microscope (Leica, TCS SP8). Fluorescence intensity was quantified using ImageJ software.

### Enzyme-linked immunosorbent assay

Hippocampal tissues were homogenized at a ratio of 1 mg tissue to 9 µL PBS, followed by thorough homogenization using an ultrasonic disruptor. The homogenates were then centrifuged at 3, 000 rpm for 20 min at room temperature. The supernatant was collected and transferred to a new centrifuge tube for storage at − 80 °C until further analysis. The levels of Aβ_42_ were quantified using an ELISA kit following the manufacturer’s instructions (Jiangsu Meimian Industry Co., Ltd., Jiangsu, China). The results were analyzed using a standard curve, and the OD450 values were recorded with a microplate reader.

### Transmission electron microscopy

Mouse hippocampal tissues were sectioned into ~ 1 mm³ blocks and fixed in 2. 5% glutaraldehyde for 1 h at room temperature, followed by 12–24 h of fixation at 4 °C. The tissues were washed three times with 0. 1 M PBS (pH 7. 2), then post-fixed in 1% osmium tetroxide for 1–2 h. After removing the fixative, tissues were washed with PBS and distilled water. Dehydration was carried out using a graded ethanol series (30%-100%) for 15 min at each concentration, followed by three changes of 100% propylene oxide. Tissue infiltration was carried out using resin and propylene oxide mixtures at a 1:1 ratio for 2 h and a 1:2 ratio overnight, followed by two rounds of incubation in pure resin at room temperature for 4 h each. Resin blocks were polymerized at 37 °C for 48 h, 45 °C for 12 h, and 60 °C for 48 h. Ultrathin Sects (60–70 nm) were prepared using an ultramicrotome, stained with uranyl acetate and lead citrate, and mounted on copper grids. Imaging was performed using a transmission electron microscope at 80 kV.

### Cell culture

HT22 cells were cultured in Dulbecco’s Modified Eagle Medium (DMEM) supplemented with 10% fetal bovine serum (FBS), 100 U/mL penicillin, and 100 µg/mL streptomycin. Cells were maintained in a humidified incubator at 37 °C with 5% CO_2_. The culture medium was replaced every two days to ensure adequate nutrient supply and remove metabolic waste. When cells reached approximately 80–90% confluence, they were either used for subsequent experiments. Small interfering RNA (siRNA) targeting HSPA1L was purchased from Hantang Bio (Shanghai, China), and transfections were carried out using Fulltrans Gold transfection reagent (Cat # FT201) at a final concentration of 2. 5 µL/mL siRNA. Transfection efficiency was assessed using CY3-labeled siRNA, as evidenced by red fluorescence observed under a fluorescence microscope (CKX 53, Olympus), and by quantitative real-time PCR (qPCR) and Western blot (WB) analysis.

### Statistical analysis

Statistical analyses were performed using GraphPad Prism 9 software. Two groups of comparisons were analyzed using a two-tailed Student’s t-test. Multiple group data were analyzed using one-way ANOVA followed by Tukey’s post hoc test. Morris water maze latency data were analyzed using two-way ANOVA followed by Šídák’s or Dunnett’s post hoc test. 0.05 was considered statistically significant.

## Results

### Multimodal intervention prevented cognitive and affective impairments in AD model mice and AD transgenic mice

Recognition memory and spatial memory deficits were early impairments of AD. Therefore, we use the new object recognition test and Morris water maze to evaluate the protective effects of multimodal intervention on recognition memory and spatial memory of AD mice. After one month of intervention, novel object recognition tests were conducted. During the training section, each mouse was allowed to explore two identical objects, no discrimination index was observed among groups, indicating that the mice did not exhibit any innate preference between the two identical objects (Fig. 1C). However, during the testing section, when one object was replaced by a novel one, AD model mice exhibited significantly lower discrimination index (*P <* 0. 001, Fig. 1D), reflecting impaired recognition memory. The decreased discrimination indices in AD model mice were reversed by exercise, 40 Hz audiovisual stimulations, as well as a combination of these interventions (*P <* 0. 01, Fig. 1D).

The Morris water maze test was used to investigate whether spatial memory was also reversed by multimodal intervention. During training, the escape latency was significantly longer in AD model mice than in WT mice (*P <* 0. 05, Fig. 1F). Notably, both single and multimodal interventions significantly reduced escape latency in AD model mice, demonstrating their effectiveness in ameliorating spatial memory deficits of AD mice (*P <* 0. 05, Fig. 1F). During the probe test, AD model mice spent significantly less time in the target quadrant and had fewer platform crossings. Multimodal intervention significantly reversed these impairments (*P <* 0. 01, Fig. 1G-H). Interestingly, the multimodal intervention resulted in significant improvement in time spent in the target quadrant compared to single interventions, suggesting an additive beneficial effect. No significant difference in swimming speed was found among groups, indicating that the observed differences were not due to motor function variability (Fig. 1I).

**Fig. 2:**
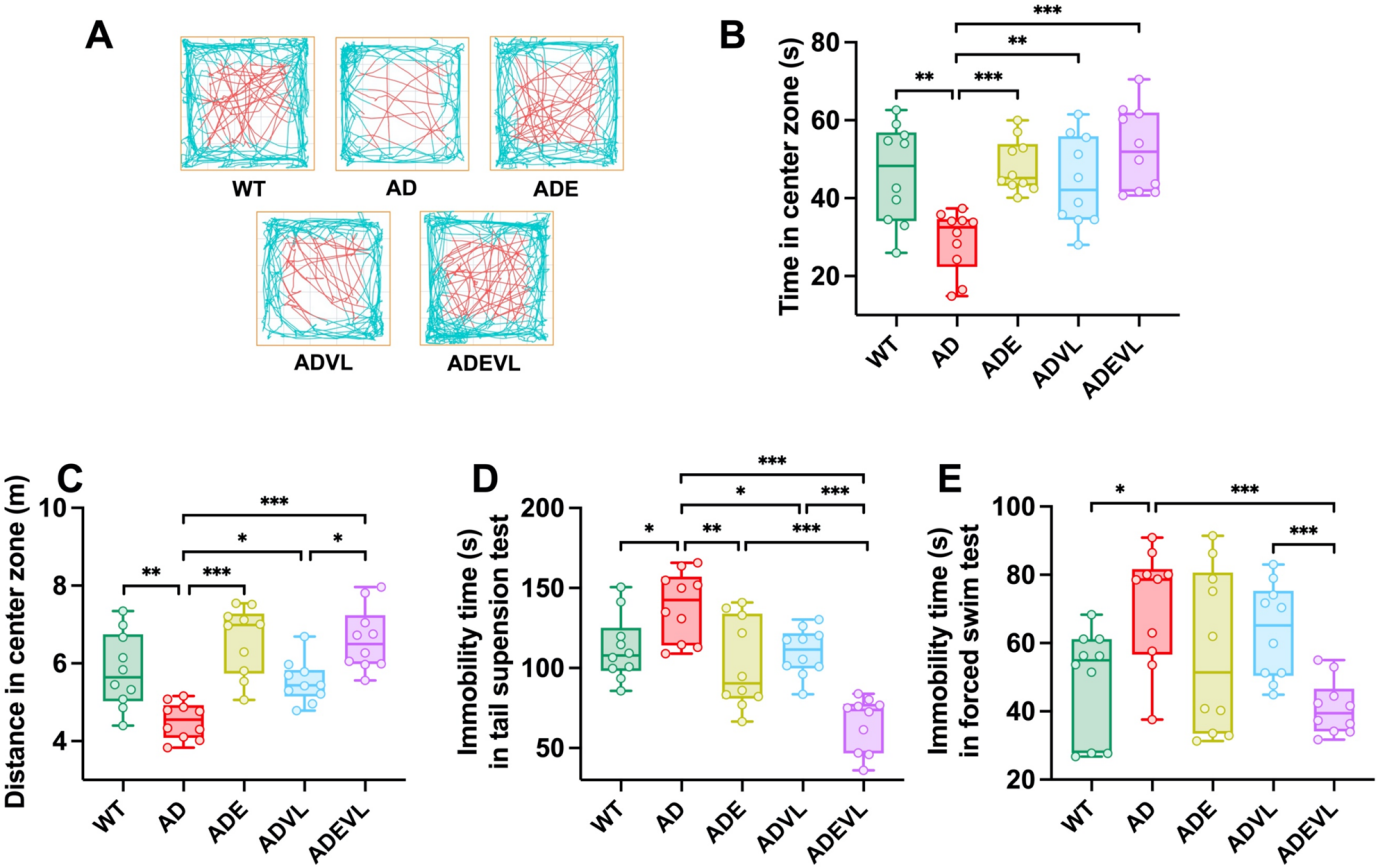
Multimodal intervention reversed the anxiety/depression-like behaviors of AD mice. **A** Representative trajectories in the open field (OF). **B** Time spent in the center zone in the OF. **C** Distance traveled in the center zone in the OF. **D** Immobility time in the tail suspension test. **E** Immobility time in the forced swim test. *n* = 10 per group, *P < 0. 05, **P < 0. 01, ***P < 0. 001 (one-way ANOVA followed by Tukey’s post hoc test)

To assess whether multimodal intervention also reversed anxiety-related behaviors of AD model mice under isolated environmental stress, we conducted open field tests. AD model mice displayed decreased activity in the central area, predominantly moving along peripheral zones, compared to controls. Significant reductions in central area exploration time and distance were observed in AD mice compared to controls (*P <* 0. 01, Fig. 2B-C). However, both single and multimodal interventions significantly reversed the decreased exploration time and distance in the central area observed in the AD model mice, suggesting that exercise and audiovisual stimulations effectively reversed anxiety-like behaviors of AD mice (*P <* 0. 01, Fig. 2B-C).

We further used the tail suspension and the forced swimming tests to investigate the beneficial effects of multimodal intervention on depression-like behaviors of AD model mice. AD model mice displayed significantly longer immobility times in both tests compared to WT mice (*P <* 0. 05, Fig. 2D-E). The single intervention significantly decreased the immobility time of AD mice in the tail suspension. However, multimodal intervention significantly reduced immobility in both the tail suspension test and the forced swimming test, suggesting that it exerted synergistic protective effects in reversing depression-like behaviors in AD mice (*P <* 0. 001, Fig. 2D-E).

**Fig. 3:**
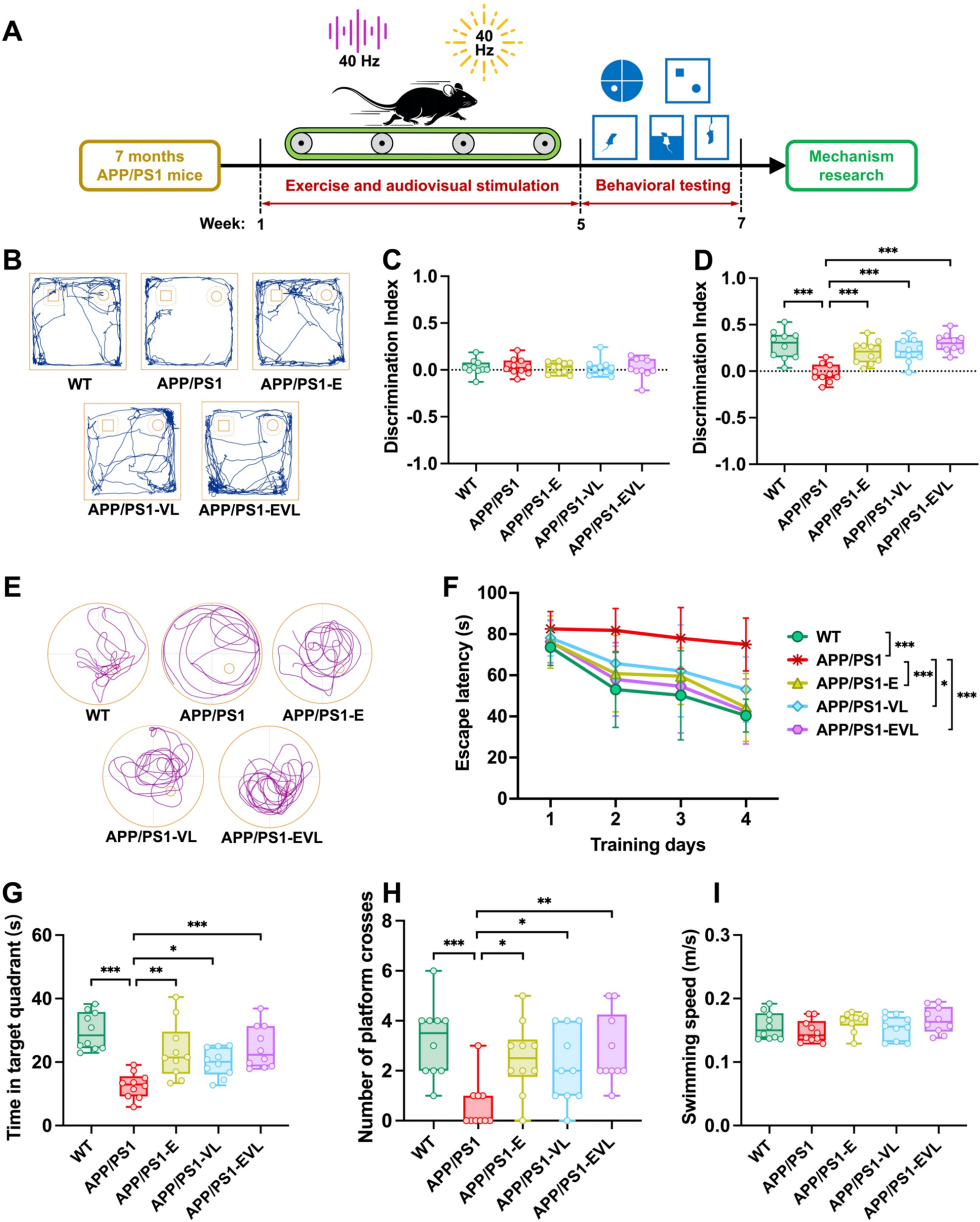
Multimodal intervention reversed the cognitive impairments of APP/PS1 transgenic mice. **A** Schematic illustration of the experimental timeline. **B** Representative trajectories during the retention session in the novel object recognition (NOR). **C** Discrimination index during the training session in the NOR. **D **Discrimination index during the retention session in the NOR. **E** Representative swimming trajectories during the spatial probe test in the Morris water maze (MWM). **F **Escape latency during spatial acquisition training in the MWM. **G** Time spent in the target quadrant during the spatial probe test in the MWM. **H **Number of target platform crosses during the spatial probe test in the MWM. **I** Swimming speed during the spatial probe test in the MWM. *n* = 10 per group, *P < 0. 05, **P < 0. 01, ***P < 0. 001 (one-way ANOVA followed by Tukey’s post hoc test or two-way ANOVA followed by Dunnett’s post hoc test)

To confirm the protective effects of multimodal intervention, we have replicated our behavioral experiments in an independent genetic model (APP/PS1 mice) (Fig. 3). Our results indicated that both recognition and spatial memory of eight-month-old APP/PS1 transgenic mice were significantly decreased, and multimodal intervention reversed cognition deficits of APP/PS1 transgenic mice (Fig. 3). Novel object recognition tests were used to assess the recognition memory of mice (Fig. 3B). During the training phase, no discrimination index was observed among groups (Fig. 3C), indicating no baseline preference between the identical objects. In the testing phase, after one object was replaced with a novel one, AD transgenic mice showed lower discrimination indices than WT controls (*P <* 0. 001, Fig. 3D), consistent with impaired recognition memory. This reduction was attenuated by exercise, 40 Hz audiovisual stimulation, and their combination (*P <* 0. 001, Fig. 3D). Overall, these findings indicate that multimodal intervention improves recognition memory performance in APP/PS1 mice.

The Morris water maze test was conducted to investigate whether the spatial learning and memory deficits of APP/PS1 mice were also reversed by multimodal intervention. During the 4-day acquisition phase, AD mice exhibited a significantly longer escape latency compared to WT mice (*P <* 0. 001, Fig. 3F), indicating a marked impairment in spatial learning. Notably, both single and multimodal interventions significantly reduced the escape latency in AD mice, demonstrating their effectiveness in ameliorating these learning deficits (*P <* 0. 05, Fig. 3F). In the subsequent probe test, AD mice spent significantly less time in the target quadrant and showed fewer platform crossings compared to the WT group (*P <* 0. 001, Fig. 3G-H). Multimodal intervention significantly reversed these spatial memory impairments (*P <* 0. 01, Fig. 3G-H). Representative swimming tracks further illustrated a clear preference for the target quadrant in the intervention groups, particularly in the EVL-treated mice (Fig. 3E). Importantly, no significant difference in swimming speed was found among all groups (Fig. 3I), confirming that the observed improvements in spatial memory were not due to variability in motor function. Together, these results demonstrate that multimodal intervention effectively alleviates spatial learning and memory deficits in the APP/PS1 mouse model.

**Fig. 4:**
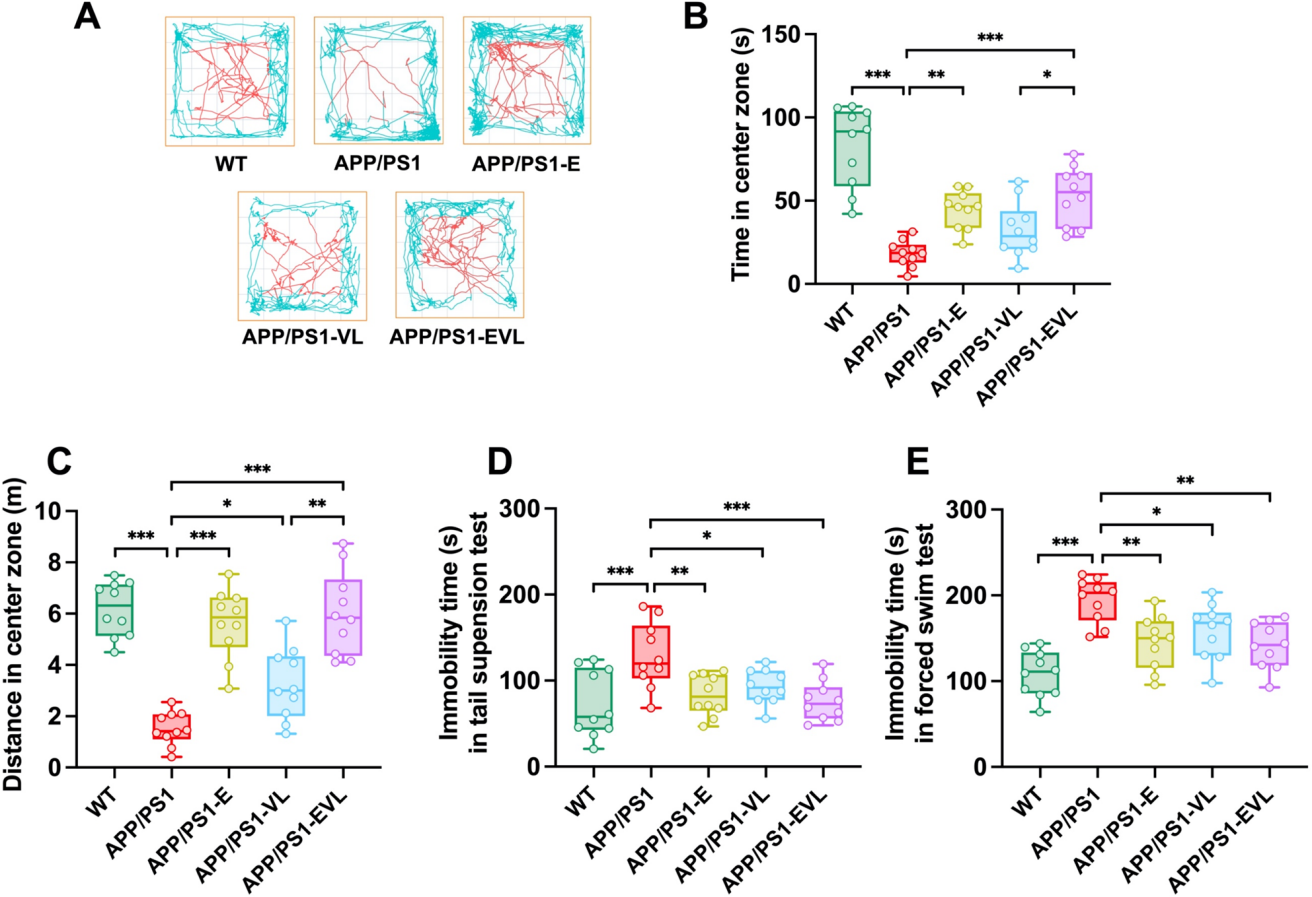
Multimodal intervention reversed the anxiety/depression-like behaviors of APP/PS1 transgenic mice. **A** Representative trajectories in the open field (OF). **B** Time spent in the center zone in the OF. **C** Distance traveled in the center zone in the OF. **D** Immobility time in the tail suspension test. **E **Immobility time in the forced swim test.* n* = 10 per group, *P < 0. 05, **P < 0. 01, ***P < 0. 001 (one-way ANOVA followed by Tukey’s post hoc test)

To assess whether multimodal intervention also reversed anxiety-related behaviors of APP/PS1 mice under isolated environmental stress, we conducted open field tests. AD mice displayed decreased activity in the central area, predominantly moving along peripheral zones compared to WT controls (Fig. 4). Significant reductions in central area exploration time and distance (*P <* 0. 001, Fig. 4B-C) were observed in AD mice compared to controls. However, both single and multimodal interventions significantly reversed the decreased exploration distance and time in the central area observed in the AD mice (Fig. 4B-C). Specifically, exercise and the multimodal intervention showed the most robust recovery effects, suggesting that exercise and audiovisual stimulation effectively reversed anxiety-like behaviors of AD mice. Notably, the multimodal intervention demonstrated a superior trend in increasing central exploration distance compared to single audiovisual stimulation (*P <* 0. 05, Fig. 4B-C), indicating an enhanced therapeutic benefit of the combined approach.

We further used tail suspension and forced swimming tests to investigate the beneficial effects of multimodal intervention on depression-like behaviors of APP/PS1 mice. AD mice displayed significantly longer immobility times in both tests compared to WT mice (*P <* 0. 001, Fig. 4D-E). Single interventions significantly decreased the immobility time of AD mice in both the forced swimming test (*P <* 0. 05, Fig. 4D) and the tail suspension test (*P <* 0. 05, Fig. 4E). Notably, the multimodal intervention significantly reduced immobility in both tests (*P <* 0. 01, Fig. 4D-E), returning the values toward homeostatic levels and suggesting that multimodal intervention exerted synergistic protective effects on reversing depression-like behaviors in AD mice.

Collectively, these findings indicated that multimodal intervention also reversed cognitive deficits and ameliorated anxiety/depression-like behaviors of AD transgenic mice.

### Multimodal intervention reduced Aβ42 accumulation, tau phosphorylation and synapse loss in AD mice

**Fig. 5:**
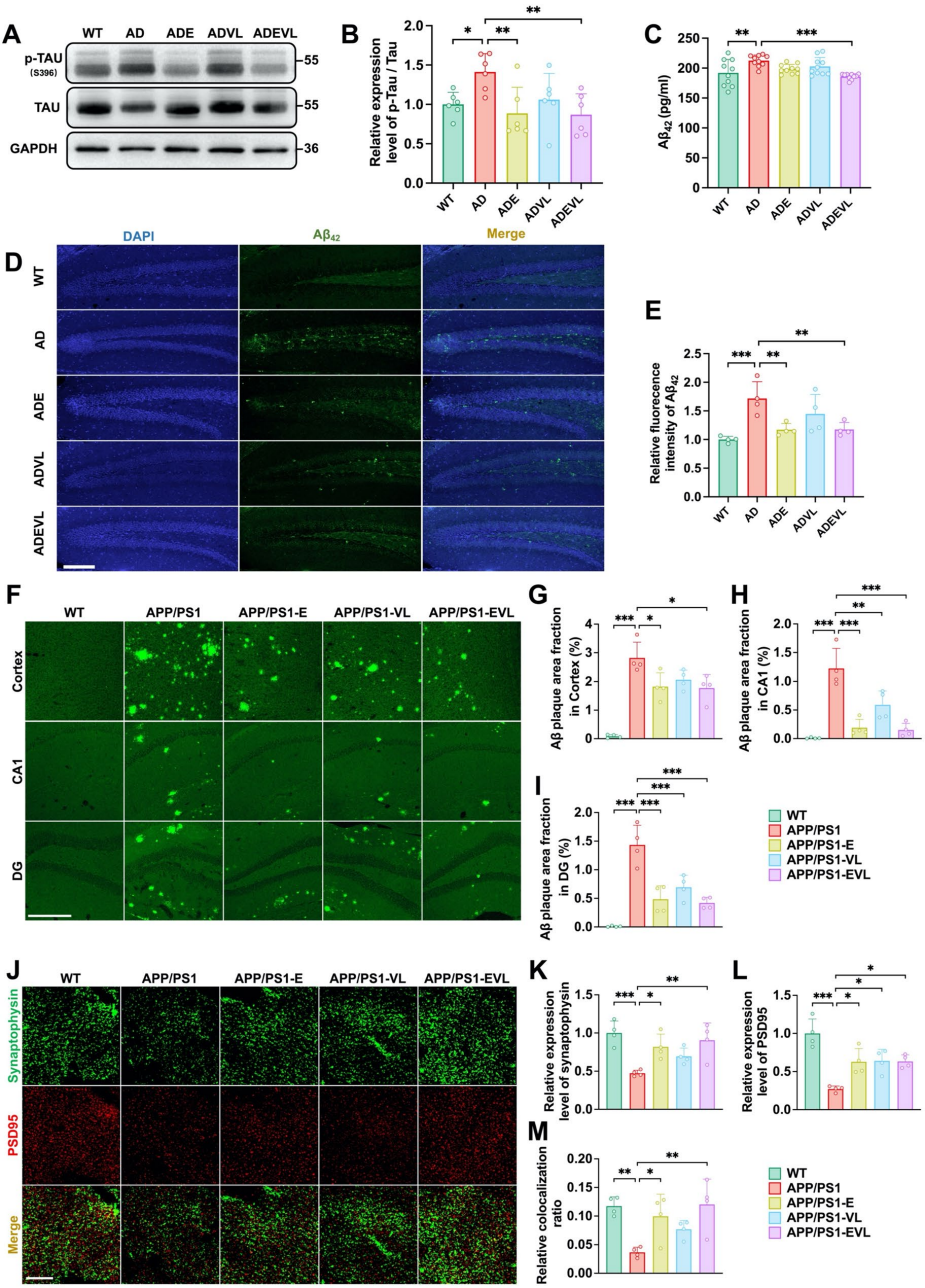
Multimodal intervention reduced Aβ deposition, tau phosphorylation and prevented synapse loss in AD mice. **A** Representative WB images of hippocampal p-Tau (S396), total Tau, and GAPDH. **B** Quantitative comparison of hippocampal p-Tau/Tau ratio in different groups. **C** Quantitative comparison of the level of hippocampal Aβ₄₂ among different groups.** D** Sample images of immunofluorescence staining of Aβ plaques in the hippocampal region among different groups (DAPI in blue, Aβ in green). Scale bar = 200 μm. **E** Quantification of Aβ fluorescence intensity. **F** Representative Thio S staining images of amyloid plaques in the cortex, hippocampal CA1, and DG regions among different groups. Scale bar = 250 μm. **G-I **Quantitative analysis of the Aβ plaque area fraction in the cortex (**G**), CA1 (**H**), and DG (**I**). **J** Representative immunofluorescence images of Synaptophysin (green) and PSD95 (red) in the hippocampal region among different groups. Scale bar =5 μm. **K-L** Quantitative comparison of the relative expression levels of Synaptophysin (**K**) and PSD95 (**L**) in the hippocampus. **M** Quantitative analysis of the colocalization ratio of Synaptophysin and PSD95. *n *= 4-10 per group, *P < 0. 05, **P < 0. 01, ***P < 0. 001 (one-way ANOVA followed by Tukey’s post hoc test)

Aβ_42_ accumulation, hyperphosphorylation of Tau protein, and synapse loss are hallmarks of the pathological features of AD. To explore the mechanisms underlying the beneficial effects of multimodal intervention on cognition and affective disorder in AD mice, both Tau hyperphosphorylation, Aβ_42_ accumulation, and synapse loss were measured. Compared with WT mice, the p-Tau/Tau protein levels were significantly elevated in AD model mice (*P <* 0. 05, Fig. 5A-B). Both exercise treatment and multimodal intervention significantly reduced hippocampal p-Tau/Tau levels in AD mice (*P <* 0. 01, Fig. 5A-B). We also assessed Aβ_42_ accumulation in the hippocampal region using immunofluorescence and ELISA. Aβ_42_ level in the hippocampus was significant increased in the AD mice (*P <* 0. 05, Fig. 5C-E). Multimodal intervention significantly reversed the increased Aβ_42_ level in the AD model mice (*P <* 0. 05, Fig. 5C-E). The beneficial effects of multimodal intervention on Aβ accumulation were also investigated in the APP/PS1 transgenic mice. Thio-S staining, which labels the cross-β-sheet structures of dense-core plaques, showed no detectable signal in WT mice. In contrast, AD mice exhibited abundant dense-core plaques in the cortex, CA1, and DG regions (*P <* 0. 001, Fig. 5F-I). Exercise and audiovisual stimulation, either alone or combined, significantly reduced Aβ plaque area in AD transgenic mice (Fig. 5G-I). To explore the role of multimodal intervention on synapse loss, we performed double immunolabeling for the presynaptic marker synaptophysin and the postsynaptic marker PSD95 in the hippocampus (Fig. 5J-M). Compared with WT mice, AD mice showed reduced colocalization of synaptophysin and PSD95, consistent with synapse loss (*P <* 0. 01, Fig. 5J-M). All intervention groups showed partial recovery relative to AD mice (Fig. 5J-M).

Together, these results demonstrated that multimodal intervention effectively reduced Aβ accumulation, Tau hyperphosphorylation and synapse loss in the hippocampus, providing mechanistic support for its protective effects on cognition and emotion in AD mice.

### Proteomics revealed that cognitive restoration following multimodal intervention was associated with the recovery of autophagy-lysosomal function and the attenuation of inflammatory factors in AD mice

**Fig. 6:**
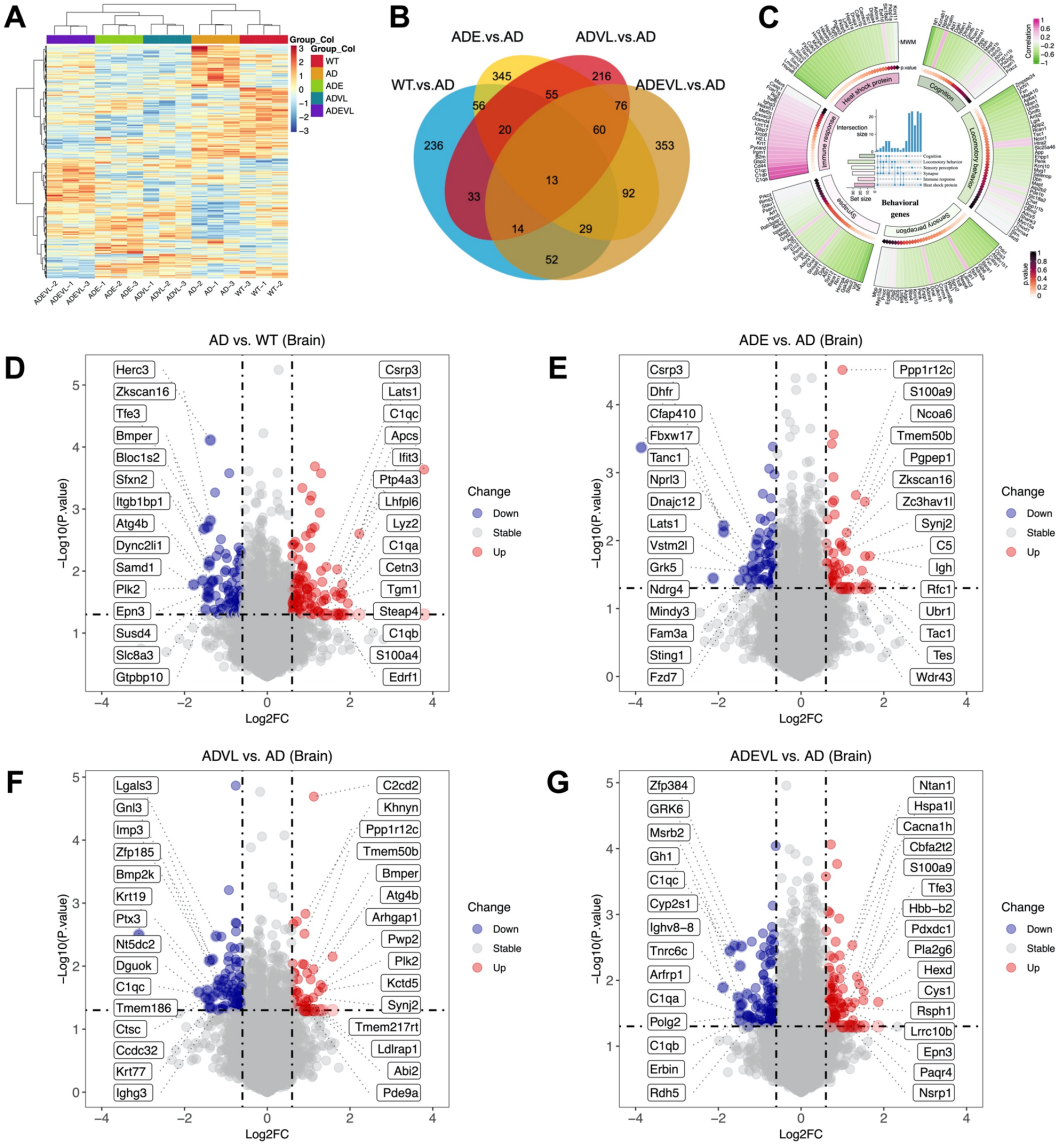
The DEPs among AD and other groups. **A** Heatmap shows distinct protein expression profiles across WT, AD, ADE, ADVL, and ADEVL groups. **B** Venn diagram illustrating unique and overlapping DEPs among other groups compared to AD. **C** Circos heatmap showing Pearson’s correlation coefficients between the average escape latency on day 4 of Morris water maze training and the expression levels of cognition, synapse, sensory perception, locomotor behavior, heat shock, and immune response related proteins of experimental groups. Warmer colors indicate positive correlations, while cooler colors represent negative correlations. **D-G** Volcano plots show significantly altered proteins in WT vs. AD D, ADE vs. AD (**E**), ADVL vs. AD (**F**), and ADEVL vs. AD (**G**) comparisons

To further investigate the molecular changes associated with AD and assess the effects of multimodal intervention, we performed quantitative proteomics. Heatmap analysis demonstrated clear segregation of protein expression profiles across experimental groups. The AD group exhibited a distinct and clustered expression pattern, markedly different from WT mice, indicative of widespread molecular disruption. The ADEVL group exhibited a molecular profile that more closely resembled that of the WT group, particularly in terms of cluster distribution, indicating that the multimodal intervention partially normalized AD-related protein dysregulation (Fig. 6A). Venn diagram analysis revealed both unique and overlapping differentially expressed proteins (DEPs) among the intervention groups compared to AD. The ADEVL group shared 92 DEPs with the ADE group and 76 DEPs with the ADVL group, while also exhibiting 353 unique DEPs that were not present in either of the single-modality groups. These findings underscore the broader molecular modulation achieved by the multimodal intervention, further supporting its potential to target diverse pathological mechanisms in AD (Fig. 6B).

To further investigate the molecular basis of cognitive recovery, we performed a Pearson’s correlation analysis between the escape latency (on the last day of MWM training) and the expression levels of representative differentially expressed proteins (DEPs). Six protein groups identified in GSEA data with significant positive or negative enrichment in key biological processes, including cognition, motor ability, sensory perception, synapses, immune response, and protein folding, were selected for correlation analysis with Morris’s test results (Fig. 6C). The Circos heatmap reveals distinct correlation profiles across the experimental groups. AD risk-related proteins like PSEN1 and PICALM, as well as immune response factors (e.g., C1qa, C1qb, Cd44), exhibited strong positive correlations with escape latency, indicating that their accumulation is intrinsically linked to impaired spatial memory. While neuroprotective factors like heat shock proteins (e.g., Hspa8, Hspa1l, Dnajc9) showed negative correlations with escape latency, indicating their protective effects in spatial memory. Circos plot provides evidence that the cognitive rescue afforded by multimodal intervention might be driven by the coordinated stabilization of the protein and the suppression of neuroinflammatory pathways.

Volcano plot analysis also revealed distinct proteomic alterations across intervention groups compared to AD controls (Fig. 6D-G). Upregulated proteins in AD mice were enriched in immune and inflammatory pathways, such as complement component 1q (C1q), reflecting excessive complement activation. Downregulated proteins included regulators of autophagy and lysosomal function, such as transcription factor e3 (TFE3) and autophagy related 4b cysteine peptidase (ATG4B) (Fig. 6D). Exercise intervention significantly decreased the expression of pro-inflammatory proteins such as stimulator of interferon genes protein 1 (STING1), while increasing transcriptional regulators such as zinc finger with krab and scan domains 16 (ZKSCAN16) and synaptic proteins such as synaptojanin 2 (SYNJ2), indicating that exercise modulates both inflammatory and neuronal pathways in the AD brain (Fig. 3E). Audiovisual stimulation profoundly diminished the expression of inflammatory-related proteins such as C1q, while significantly upregulating autophagy-linked proteins such as ATG4B (Fig. 6F). Notably, Multimodal intervention efficiently reversed the upregulation of inflammatory proteins in AD mice, such as C1q, while promoting the expression of lysosomal and autophagic regulators such as TFE3 (Fig. 6G). These results demonstrated the broad yet selective capacity of multimodal intervention to reverse AD-associated protein dysregulation and restore key cellular pathways critical for immune homeostasis and protein clearance.

**Fig. 7:**
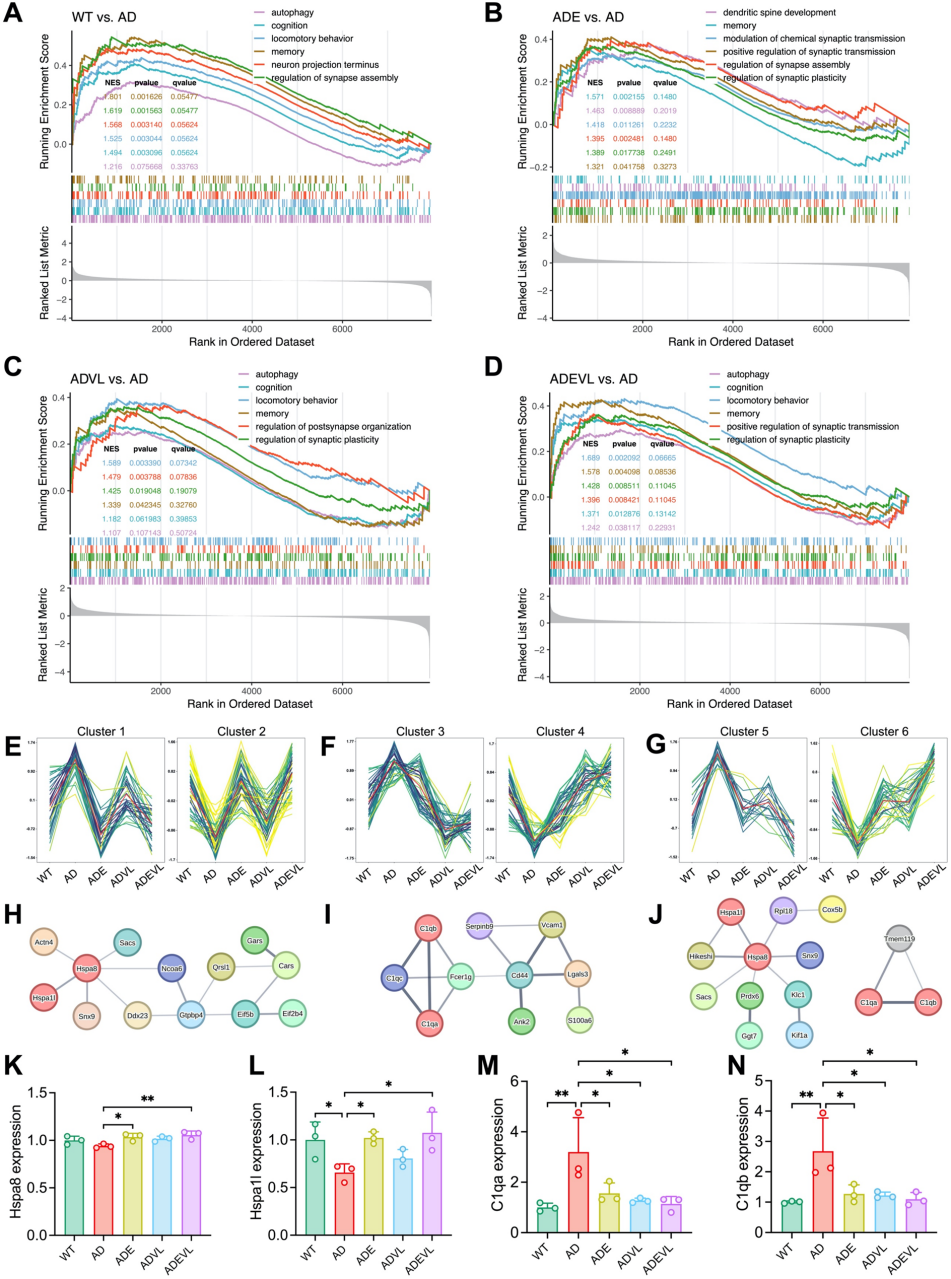
Multimodal intervention reinforced the network of auto-lysosomal and reduced pro-inflammatory factors. **A** GSEA of WT vs. AD shows downregulation of autophagy, cognition, locomotor behavior, memory, neuron projection terminus, and synapse assembly in AD. **B** GSEA of ADE vs. AD shows enrichment of dendritic spine development, memory, synaptic transmission, synapse assembly, and synaptic plasticity in the ADE group. **C** GSEA of ADVL vs. AD shows enrichment of autophagy, cognition, locomotor behavior, memory, postsynapse organization, and synaptic plasticity in the ADVL group. **D** GSEA of ADEVL vs. AD demonstrates the strongest restoration of autophagy, cognition, locomotor behavior, memory, positive regulation of synaptic transmission, and synaptic plasticity by the multimodal intervention. **E** Trend clustering of DEPs showing exercise advantage restoration, and the red line is the overall trend line.** F** Trend clustering of DEPs showing audiovisual stimulation advantage restoration. **G** Trend clustering of DEPs showing multimodal intervention advantage restoration. **H** The PPI network of DEPs identified in the exercise advantage cluster. **I** The PPI network of DEPs identified in the audiovisual stimulation favored advantage cluster.** J** The PPI network of DEPs identified in the multimodal intervention advantage cluster.** K-N** Quantitative proteomic expression of Hspa8, Hspa1l, C1qa, and C1qb in each group.* n* = 3 per group, *P < 0. 05, **P < 0. 01, ***P < 0. 001 (one-way ANOVA followed by Tukey’s post hoc test)

Gene set enrichment analysis (GSEA) revealed profound disruptions induced by AD in pathways related to autophagy, cognition, locomotor behavior, memory, and synaptic assembly, as demonstrated by significantly diminished enrichment compared with WT controls (Fig. 7A). In the ADE group, mild positive enrichment was observed predominantly in synaptic development and plasticity pathways (Fig. 7B). In the ADVL group exhibited positive enrichment in multiple pathways involving autophagy, cognitive processes, memory, and synaptic function (Fig. 7C). Importantly, the ADEVL group demonstrated the most robust and comprehensive enrichment across all examined pathways, closely resembling the gene activation profiles observed in WT mice (Fig. 7D). Collectively, these results underscored the enhanced efficacy of multimodal intervention in concurrently improving autophagic and cognitive pathways compromised in AD.

To examine how the multimodal intervention restores AD-related pathways and to find out the differences and synergies between the individual and combined therapies, we conducted trend clustering along with protein–protein interaction (PPI) network analysis to characterize the molecular features of each treatment. The clusters 1 and 2 showed an exercise-dominant pattern (Fig. 7E). The corresponding PPI network (Fig. 7H) was centered on molecular chaperones (HSPA8/HSPA1L), dynamic lysosome transport proteins [Sacsin Molecular Chaperone (Sacs)] and protein translation-related factor [Eukaryotic Translation Initiation Factor 5B (Eif5b)]. This pattern suggests that exercise might regulate protein synthesis especially stress-related protein to restore proteostasis and promoting the clearance of protein aggregates via the autophagy-lysosomal pathway. In contrast, clusters 3 and 4 reflected audiovisual stimulation advantage restoration effects (Fig. 7F). Its PPI network (Fig. 7I) formed a tightly connected neuroimmune module including complement components (C1qa, C1qb, and C1qc), immune cell migration related protein [CD44 Molecule (CD44)], vascular cell adhesion protein [Vascular Cell Adhesion Molecule 1 (Vcam1)] and calcium-binding protein [Calcium Binding Protein A6 (S100a6)]. These results indicate that audiovisual intervention mainly acts by modulating complement-driven immune responses and neuroinflammatory signaling. Clusters 5 and 6 captured the proteins that might have synergistic effects of exercise and audiovisual intervention (Fig. 7G), and identified genes for which the combined treatment outperformed single interventions. Stress-related protein (HSPA8/HSPA1L) and complement factors (C1qa/C1qb) are hub gene in clusters 5 and cluster 6, respectively (Fig. 7J). Consistent with these network-level observations, quantitative analysis showed that the decreased expression of HSPA8/HSPA1L and the elevated expression of C1qa/C1qb in AD mice were effectively reversed by the multimodal intervention (Fig. 7K-N).

Collectively, our proteomic findings indicated that multimodal intervention not only globally remodeled the protein expression profile in AD mice but also explicitly reinforced the network of auto-lysosomal regulators and reduced pro-inflammatory factors.

### Multimodal intervention reduced neuroinflammation in the hippocampus of AD mice

**Fig. 8:**
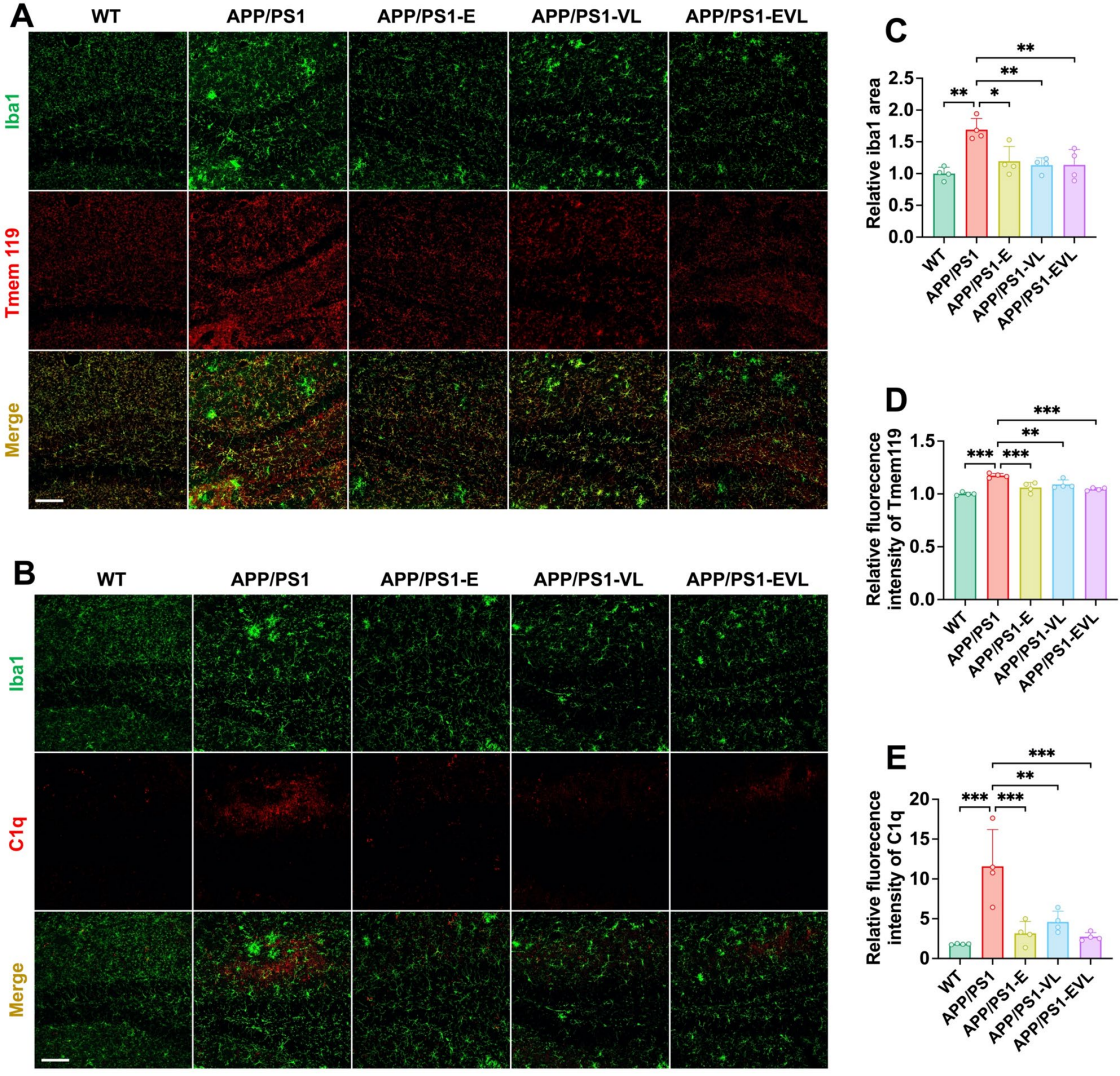
Multimodal intervention significantly decreased microglia activation and neuroinflammatory reaction in AD Mice. **A** Sample images of immunofluorescence staining of Iba1 and Tmem119 in the hippocampal region among different groups (Iba1 in green, Tmem119 in red). **B** Sample images of immunofluorescence staining of Iba1 and C1q in the hippocampal region among different groups (Iba1 in green, C1q in red). **C** Quantitative analysis of the Iba1 area. **D** Quantitative analysis of the Tmem119 fluorescence intensity. **E** Quantitative analysis of the C1q fluorescence intensity. Scale bar = 100 μm, n = 4 per group, *P < 0. 05, **P < 0. 01, ***P < 0. 001 (one-way ANOVA followed by Tukey’s post hoc test)

To validate that multimodal intervention exerts its protective effect through reducing neuroinflammation as identified by proteomic analysis, we performed dual immunofluorescence staining for Tmem119 / C1q together with the microglial marker Iba1. In the hippocampus of APP/PS1 mice, the Iba1-positive area was increased compared with WT (*P <* 0. 01, Fig. 8A-C), indicating microglial activation. This was accompanied by higher levels of Tmem119 and C1q. Tmem119 fluorescence intensity was markedly elevated in AD mice (*P <* 0. 01, Fig. 8D), and C1q showed strong immunoreactivity, largely localized around activated microglia (*P <* 0. 001, Fig. 8B, E). Multimodal intervention attenuated these changes in AD mice (*P <* 0. 01, Fig. 8C, D, E). With the multimodal intervention, the Iba1-positive area was reduced in AD mice (*P <* 0. 05, Fig. 8C), and both Tmem119 and C1q levels were decreased, approaching WT levels (*P <* 0. 001, Fig. 8D-E). Representative images show that the clustered Tmem119 and C1q signals seen in AD mice were decreased after intervention. Together, these data support the proteomic results and indicate that modulation of microglial activation is associated with the neuroprotective effects of multimodal intervention.

### Multimodal intervention exerts its beneficial effects via regulating the degradation function of lysosome

**Fig. 9:**
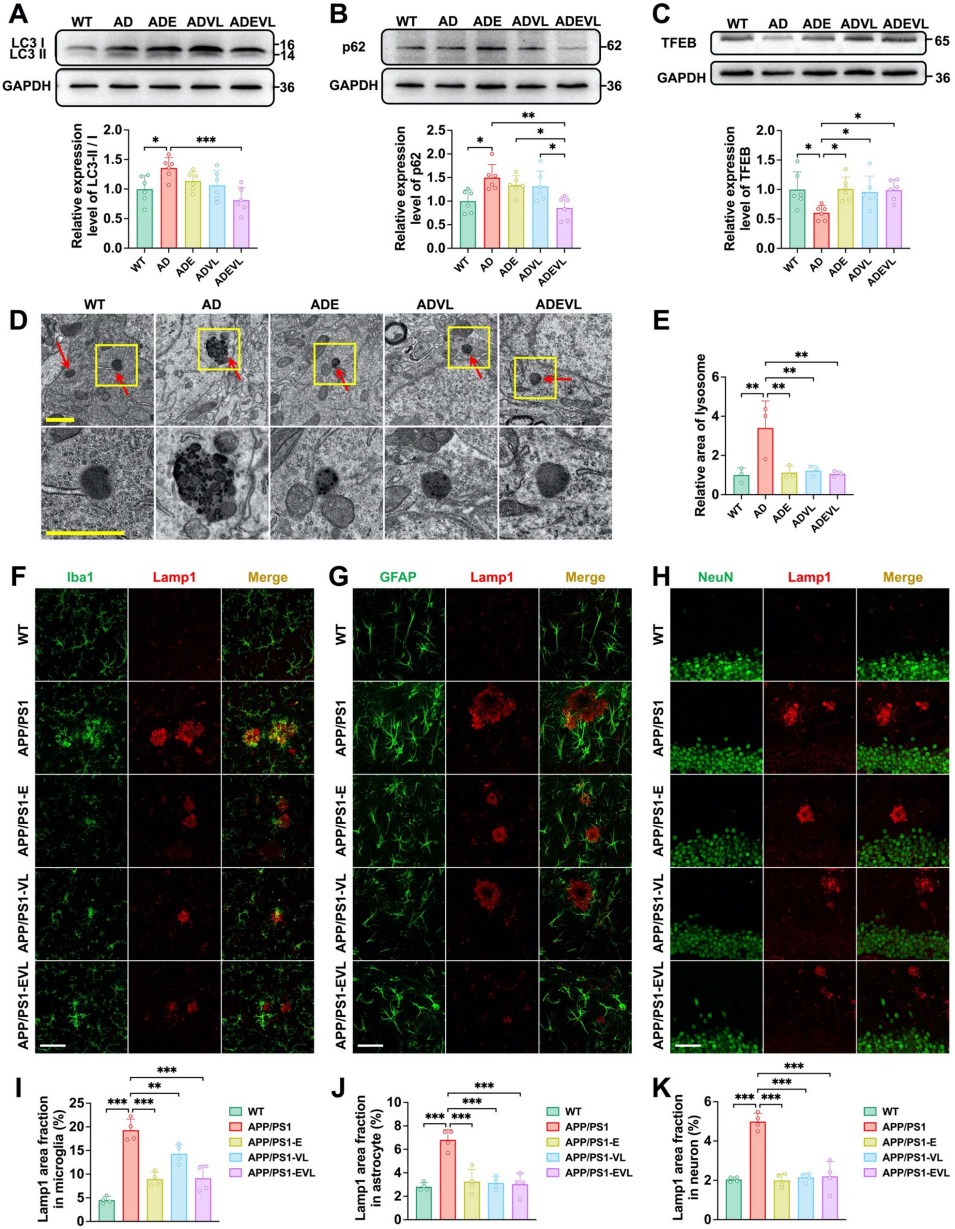
Multimodal intervention significantly improves autophagy-lysosomal system function. **A** Quantitative comparison of hippocampal LC3II/I level in different groups. **B** Quantitative comparison of hippocampal p62 level in different groups. **C** Quantitative comparison of hippocampal TFEB level in different groups. **D** TEM images showing hippocampal lysosomal morphology (red arrows). Scale bar = 1.0 μm. **E** Quantitative comparison of lysosomal area in hippocampal neurons in different groups. **F** Representative immunofluorescence images of Iba1 (green) and Lamp1 (red) in the hippocampal region among different groups. Scale bar = 50 μm. **G** Representative immunofluorescence images of GFAP (green) and Lamp1 (red) in the hippocampal region among different groups. Scale bar = 50 μm. **H** Representative immunofluorescence images of NeuN (green) and Lamp1 (red) in the hippocampal region among different groups. Scale bar = 50 μm. **I** Quantitative analysis of the proportion of Lamp1 area in microglia. **J** Quantitative analysis of the proportion of Lamp1 area in astrocytes. **K** Quantitative analysis of the proportion of Lamp1 area in neurons. *n* = 3-6 per group *P < 0. 05, **P < 0. 01, ***P < 0. 001 (one-way ANOVA followed by Tukey’s post hoc test)

Maintaining lysosomal homeostasis is crucial for the efficient degradation function of the lysosome. We therefore investigated whether a multimodal intervention could enhance lysosomal degradation capacity. We measured the ratio of LC3II/I across experimental groups. This ratio was significantly elevated in AD mice compared to WT controls. Single interventions exhibited partial effects, whereas the multimodal intervention significantly decreased the ratio of LC3II/I (*P <* 0. 001, Fig. 9A). An increased LC3II/I ratio might be caused by decreased lysosomal degradation of autophagosomes. To verify these results, we further assessed p62 expression. p62 is a substrate specifically degraded by lysosomes, whose accumulation indicates compromised lysosomal degradation function. The expression level of p62 was markedly increased in AD mice. Multimodal intervention robustly decreased p62 expression, demonstrating enhanced lysosomal degradation activity following multimodal intervention (*P <* 0. 01, Fig. 9B). Decreased lysosomal degradation in AD might be caused by decreasing lysosomal biogenesis. To determine the role of multimodal intervention in lysosomal biogenesis, we evaluated the expression of TFEB. AD mice exhibited notably lower TFEB levels than WT mice. Importantly, both the single treatment and the multimodal intervention significantly increased TFEB expression (*P <* 0. 05, Fig. 9C).

Impaired lysosomal degradation capacity might lead to structural alterations, including enlargement of lysosomes due to accumulation of undegraded material. To examine this, we used transmission electron microscopy (TEM) to analyze the lysosomal morphology. Lysosomes in AD mice appeared prominently enlarged. Conversely, lysosomes in multimodal intervention-treated mice displayed smaller size and normal morphology. The quantitative analysis revealed a significant reduction in the lysosomal area following the multimodal intervention, reinforcing the improvement in lysosomal degradation capability (*P <* 0. 01, Fig. 9D-E). To find out the cell-specific changes of lysosomes in response to multimodal intervention, we quantified the expression of lysosomal marker Lamp1 in microglia, astrocytes, and neurons in the hippocampus of AD and /or multimodal intervention-treated mice (Fig. 9F-H). AD mice exhibited a marked increase in Lamp1 area fraction compared with WT in all three types of cells, including microglia, astrocytes, and neurons (*P <* 0. 001, Fig. 9I-K). This pathological change was most pronounced in microglia, where the Lamp1 area fraction reached approximately 20% of microglia area, far exceeding the alterations observed in GFA*P-*positive astrocytes or NeuN-positive neurons. Moreover, the dense clusters of Lamp1-positive puncta were found in activated microglia in the AD group. Both single and combined interventions effectively reduced Lamp1 area in microglia, astrocytes and neurons (*P <* 0. 001, Fig. 9I-K).

Collectively, these findings show that multimodal intervention improves lysosomal function and alleviates structural alterations in the AD hippocampus. It reduces the expansion of the lysosomal compartment, particularly in microglia, and helps restore lysosomal balance, which might be associated with enhanced clearance of autophagic substrates.

### Multimodal intervention restores lysosomal membrane integrity

**Fig. 10:**
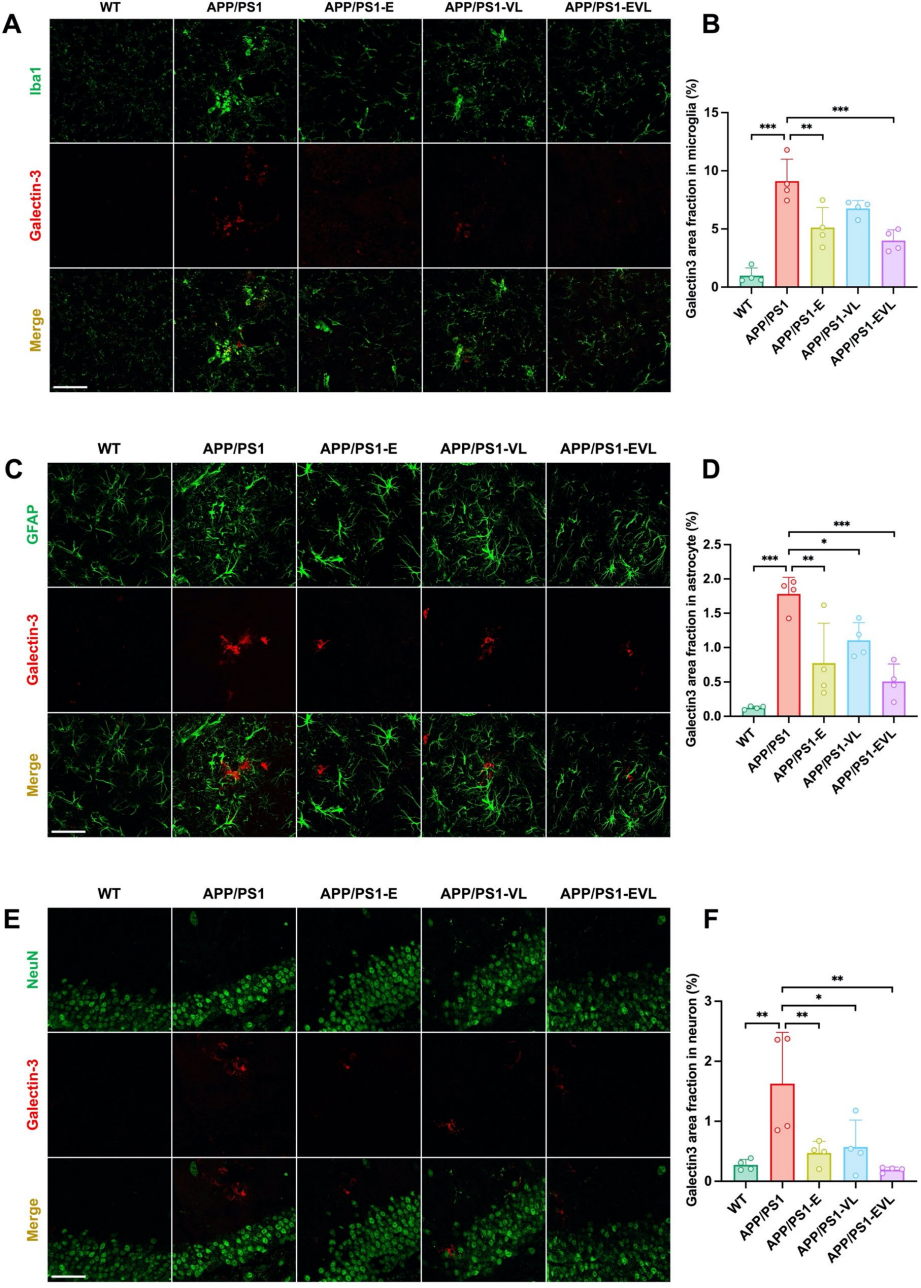
Multimodal intervention improves the lysosomal membrane integrity of AD transgenic mice. **A** Representative immunofluorescence images of Iba1 (green) and Galectin-3 (red) in the hippocampal region among different groups. **B **Quantitative analysis of the proportion of Galectin-3 area in microglia. **C** Representative immunofluorescence images of GFAP (green) and Galectin-3 (red) in the hippocampal region among different groups. **D** Quantitative analysis of the proportion of Galectin-3 area in astrocytes. **E** Representative immunofluorescence images of NeuN (green) and Galectin-3 (red) in the hippocampal region among different groups. **F** Quantitative analysis of the proportion of Galectin-3 area in neurons. Scale bar = 50 μm. *n* = 4 per group, *P < 0. 05, **P < 0. 01, ***P < 0. 001 (one-way ANOVA followed by Tukey’s post hoc test)

Lysosomal membrane integrity is essential for lysosome function. To find out whether multimodal intervention exerts its protective effects via restoring lysosomal membrane integrity, the aggregation of Gal3 was analyzed in AD transgenic mice with or without multimodal intervention treatment (Fig. 10). Quantitative analysis revealed that Gal3 levels were increased in neurons, astrocytes, and microglia in AD transgenic mice, yet this pathology was most prominent within the microglial population (*P <* 0. 001, Fig. 10). The accumulation of Gal3 in Iba1-positive cells was more severe than in astrocytes or neurons. Notably, multimodal intervention significantly reduced Gal3 area fraction (*P <* 0. 001, Fig. 10), indicating a robust improvement in lysosomal membrane stability.

### Multimodal intervention alleviates lysosomal permeability and cognitive deficits via HSPA1L

**Fig. 11:**
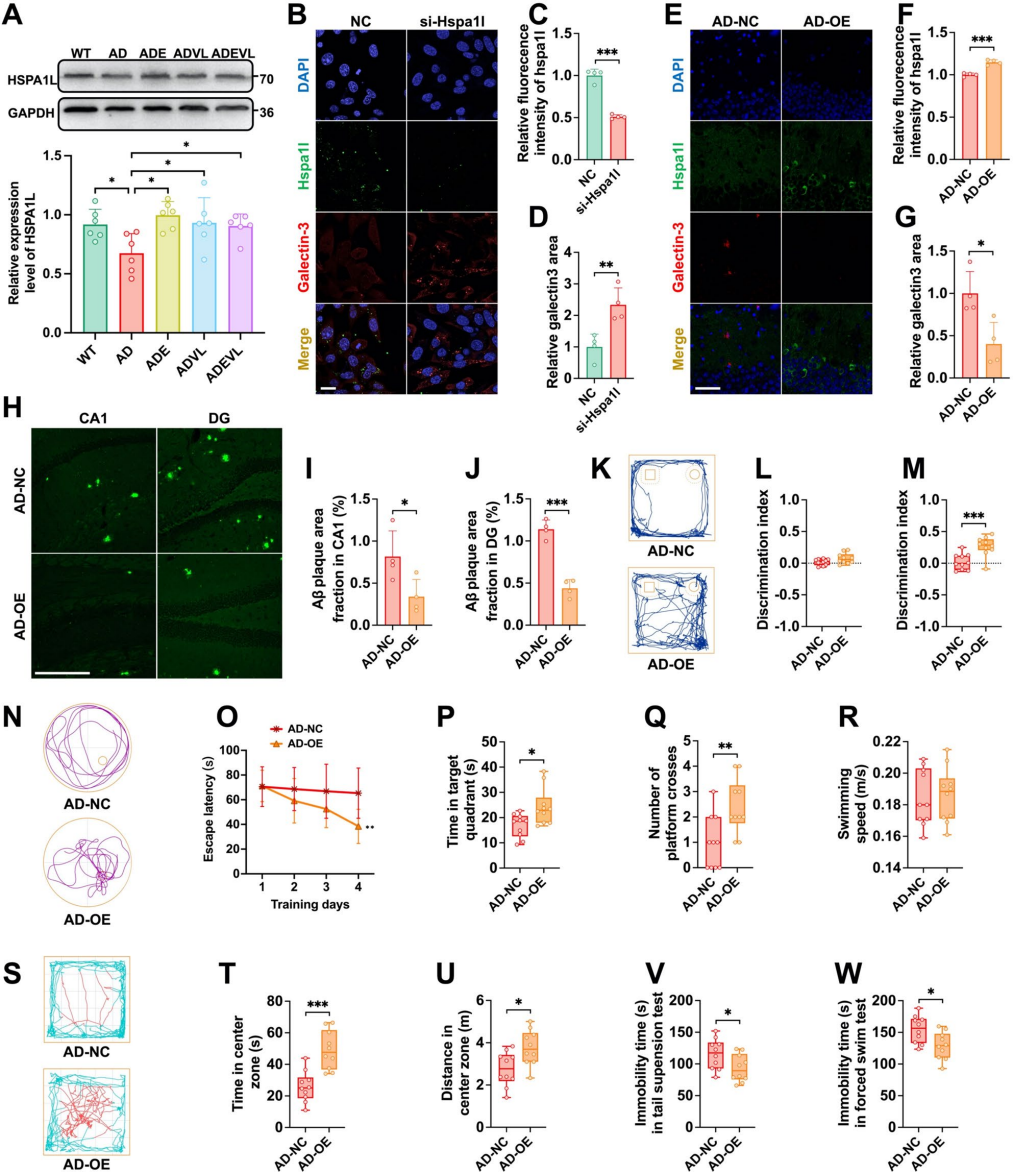
Multimodal intervention alleviates lysosomal permeability and cognitive deficits via HSPA1L. **A **Quantitative comparison of hippocampal HSPA1L level in different groups. **B** Immunofluorescence staining of Hspa1l (green), Galectin-3 (red), and DAPI (blue) in NC or si-HSPA1L-treated cells. Scale bar = 20 μm. **C-D** Quantitative analysis of Hspa1l (**C**) and Galectin-3 (**D**) fluorescence intensity in different groups. **E** Immunofluorescence staining of Hspa1l (green), Galectin-3 (red), and DAPI (blue) in AD-NC mice or AD-OE mice. Scale bar = 50 μm. **F-G** Quantitative comparison of Hspa1l (**F**) and Galectin-3 (**G**) fluorescence intensity in different groups. H Representative Thio S staining images of amyloid plaques in hippocampal CA1 and DG regions across different groups. Scale bar = 250 μm. **I-J** Quantitative analysis of the Aβ plaques area fraction in the CA1 (**I**) and DG (**J**). K Representative trajectories during the retention session in the novel object recognition (NOR).** L** Discrimination index during the training session in the NOR. **M** Discrimination index during the retention session in the NOR. N Representative swimming trajectories during the spatial probe test in the Morris water maze (**MWM**). **O** Escape latency during spatial acquisition training in the MWM. **P** Time spent in the target quadrant during the spatial probe test in the MWM. **Q** Number of target platform crosses during the spatial probe test in the MWM. **R** Swimming speed during the spatial probe test in the MWM.** S** Representative trajectories in the open field (**OF**).** T** Time spent in the center zone in the **OF**. **U** Distance traveled in the center zone in the **OF**.** V** Immobility time in the tail suspension test. **W** Immobility time in the forced swim test. *n* = 4-10 per group, *P < 0. 05, **P < 0. 01, ***P < 0. 001 (unpaired two-tailed t-test or two-way ANOVA followed by Šídák’s post hoc test)

As revealed by the proteomics, HSPA1L is the hub protein, and its expression was markedly restored following multimodal intervention. To determine whether a multimodal intervention regulating lysosomal integrity and degradation in AD mice via HSPA1L, both downregulation and upregulation of HSPA1L were used. A significant reduction in HSPA1L expression was observed in AD mice compared with WT mice (*P <* 0. 05, Fig. 11A). Multimodal intervention markedly reversed this reduction, restoring HSPA1L expression to levels comparable to those in WT mice (0. 05, Fig. 11A). To further establish the relationship between HSPA1L and lysosomal membrane integrity, we performed siRNA-mediated knockdown experiments in HT22 hippocampal neuronal cells (Fig. 11B). HSPA1L expression was significantly reduced following siRNA transfection (*P <* 0. 001, Fig. 11C). HSPA1L knockdown led to increased galectin-3 punctate structures, indicative of enhanced lysosomal membrane permeability (LMP) (*P <* 0. 01, Fig. 11D ), while upregulation of HSPA1L reduced Gal3 accumulation of AD mice (*P <* 0. 05, Fig. 11E-G).

To find out whether HSPA1L will also regulate amyloid accumulation, the thio S staining of Aβ plaques in the hippocampus of the AD transgenic mice with or without the upregulation of HSPA1L (Fig. 11H). Quantitative analysis showed that upregulation of HSPA1L significantly decreases Aβ plaque area fraction across the hippocampus of AD transgenic mice (*P <* 0. 05, Fig. 11I-J).

To assess whether upregulation of HSPA1L will reverse the cognitive deficit of AD mice, we performed the novel object test and Morris water maze test in the APP/PS1 AD transgenic mice with or without the upregulation of HSPA1L. Overexpression of HSPA1L significantly reversed the recognition memory and spatial memory of APP/PS1 AD transgenic mice (*P <* 0. 05, Fig. 11K-W). In the novel object test, during the training phase, the discrimination index did not differ between the AD-NC and AD-OE groups (Fig. 11L), indicating no baseline preference for the identical objects. In the testing phase, when one object was replaced with a novel one, the AD-OE group showed a higher discrimination index than the AD-NC group (*P <* 0. 001, Fig. 11M). In the Morris water maze test, upregulation of HSPA1L reversed the increased escape latency of the AD mice (*P <* 0. 01, Fig. 11O). In the probe test, HSPA1L-unregulated AD mice spent more time in the target quadrant (*P <* 0. 05, Fig. 11P) and showed more platform crossings than AD-NC mice (*P <* 0. 01, Fig. 11Q). Swimming speed did not differ between groups (Fig. 11R), indicating that these effects were not due to changes in locomotion. These results indicate that upregulation of HSPA1L improved spatial memory in APP/PS1 mice.

To assess whether upregulation of HSPA1L will also reverse anxiety/depression-like behaviors of AD mice, we performed open field tests, tail suspension tests, and forced swimming tests in the APP/PS1 AD transgenic mice with or without the upregulation of HSPA1L. Overexpression of HSPA1L significantly ameliorated the anxiety/depression-like behaviors of APP/PS1 AD transgenic mice (*P <* 0. 05, Fig. 11S-W). In the open field tests, HSPA1L overexpression mice spent more time in the center zone (*P <* 0. 001, Fig. 11T) and traveled a longer distance within the central area (*P <* 0. 05, Fig. 11U). During tail suspension tests and forced swimming tests, the AD-OE group showed reduced immobility time compared with the AD-NC group (*P <* 0. 05, Fig. 11V-W). These results indicate that upregulation of HSPA1L ameliorated anxiety- and depression-like behaviors of APP/PS1 mice.

These results confirmed that HSPA1L is the hub gene through which multimodal intervention maintains the integrity of the lysosomal membrane and improves lysosomal degradation function, thereby reversing cognitive impairment and ameliorating anxiety/depression-like behaviors in APP/PS1 AD transgenic mice.

## Discussion

With the rapid growth of the global aging population, AD poses a serious threat to human health [1–3]. Non-pharmacological interventions, including exercise and 40 Hz gamma audiovisual stimulation due to their broad applicability and fewer adverse effects, have emerged as promising alternatives to conventional therapies [12, 15, 29]. Our results demonstrate that multimodal intervention significantly alleviated cognitive impairment and anxiety/depression-like behaviors in both Aβ oligomer-induced and APP/PS1 transgenic mouse models. Notably, the multimodal approach exhibited partial synergistic effects compared to single-factor interventions. These results were consistent with previous studies, which indicated that multimodal interventions hold promise for achieving superior treatment outcomes in AD [30, 31].

Multimodal intervention might exert its protective effects through reducing the elevated hippocampal Aβ_42_ and p-Tau levels in AD mice. Severe cognitive and affective impairments correlate strongly with hallmark pathological features of AD, including Aβ aggregation and Tau hyperphosphorylation [4, 32, 33]. Our results indicated that multimodal intervention can effectively inhibit the accumulation of Aβ_42_ and p-Tau in the brains of AD mice, thereby improving cognitive dysfunction.

To comprehensively elucidate the mechanisms underlying the observed benefits of multimodal intervention, we further performed quantitative proteomic analysis of hippocampal tissue, revealing global remodeling of pathways involved in autophagy, synaptic function, and immune regulation. Proteomic analysis showed that multimodal intervention induced broad molecular remodeling in key pathways disrupted in AD, notably restoring many proteins associated with autophagy-lysosomal homeostasis, synaptic function, and neuroimmune signaling. These results indicate that the benefits of multimodal treatment are not limited to isolated targets but reflect a systems-level rebalancing of multiple interconnected biological processes underlying cognitive decline in AD. In addition, compared to single-modality interventions, the multimodal intervention regulated a greater number of DEPs and significantly enriched more functional pathways. This systems-level normalization encompasses a broad array of biological processes, suggesting that multimodal intervention may be more effective in addressing the multifactorial nature of AD [31, 34]. Notably, proteins associated with both immune/inflammatory and autophagy-lysosomal processes regulation exhibited the most significant recovery in the multimodal intervention group.

Furthermore, we combined trend clustering with PPI network analysis to examine potential synergy of different interventions in AD-related pathways. The PPI network of exercise-domiant pattern was centered on molecular chaperones (HSPA8/HSPA1L), dynamic lysosome transportion proteins (Sacs) and protein translation related factor (Eif5b), HSPA8 and HSPA1L are essential for recognizing and refolding misfolded proteins and maintaining protein homeostasis [35, 36]. Sacs, as key proteins involved in lysosomal dynamic transport, play an important role in organelle transport and the maintenance of autophagic flux [37], while Eif5b, as a translation initiation factor, participates in the regulation of the initiation stage of protein synthesis, has be reported to regulate the translation efficiency of specific proteins under stress conditions [38]. Thus, exercise may exert a neuroprotective effect by enhancing the synthesis and folding capacity of stress-related proteins, restoring protein homeostasis, and promoting the clearance of abnormal proteins. The PPI network of the audiovisual stimulation advantage restoration forms a highly connected neuroimmune regulatory module including complement components (C1qa, C1qb, and C1qc), immune cell migration related protein (CD44), vascular cell adhesion protein (Vcam1) and calcium-binding protein (S100a6). C1qa, C1qb, and C1qc, as initiating molecules of the classical complement pathway, play crucial roles in synaptic pruning and neuroinflammation [39], CD44 participates in inflammatory responses and immune cell migration [40], Vcam1 mediates the recruitment of peripheral immune cells across the blood-brain barrier and is closely related to neuroinflammation [41], S100a6 participates in the regulation of cellular stress and inflammatory signals [42]. Our results indicated that audiovisual stimulation significantly downregulats these proteins, thereby reversing AD neuroimmunological imbalances. This result is consistent with previous studies that 40 Hz gamma stimulation will improve microglia function and reduce Aβ deposition in AD mice [15, 16]. Abnormal protein accumulation can activate the complement system and microglia-mediated inflammatory responses, while the inflammatory environment further inhibits protein folding and clearance, forming a vicious cycle [43]. The combined intervention of the two may break the positive feedback loop of “protein misfolding-neuroinflammation”, thereby achieving a more comprehensive intervention on AD pathology.

Autophagy-lysosomal dysfunction has been recognized as an early event in the pathogenesis of AD [44]. The autophagy-lysosomal system is essential for maintaining neuronal proteostasis by clearing misfolded proteins [45]. Dysregulation of the autophagy-lysosomal system impairs the intracellular clearance of toxic aggregates such as Aβ and p-Tau, thereby contributing to AD progression [46–48]. In this study, significant downregulation of autophagy-lysosomal pathway-related factors in AD mice were found, whereas multimodal intervention markedly reversed these changes. Our results demonstrated that multimodal intervention normalizes the LC3II/I ratio and reduces p62 accumulation in AD, indicating improved clearance of autophagic substrates and restoration of degradative function. Mounting evidence indicates that neuroinflammation and impaired proteostasis are mutually reinforcing processes in AD pathology. Dysfunction of the autophagy-lysosomal system not only leads to the accumulation of pathogenic proteins such as Aβ and p-Tau, but also triggers neuroinflammatory cascades, further exacerbating neuronal injury [49]. Restoring autophagy-lysosomal function is increasingly recognized as a prerequisite for promoting the degradation of toxic protein aggregates, inhibiting neuroinflammation, and ameliorating AD pathology [50]. Our results indicated that multimodal intervention not only improved the degradation function of the lysosome but also significantly downregulated inflammatory factors in AD mice, with a particularly notable decrease in the expression levels of the membrane core complement factors C1qa and C1qb. The complement system, especially the C1q complex, has been shown to be markedly upregulated in the brains of AD models and is closely associated with synaptic damage and neuronal loss [51]. Therefore, multimodal intervention may effectively modulate neuronal damage resulting from the reciprocal promotion between autophagy-lysosomal dysfunction and neuroinflammatory activation in AD.

Multimodal intervention might improve the auto-lysosome function through increasing the biogenesis of auto-lysosomes as well as preserving the structural integrity of lysosomes. The expressions of TFEB of the AD mice were significantly increased by multimodal intervention. TFEB are critical for the biogenesis of lysosomes [52–55]. The restoration of TFEB underscores the potential of multimodal intervention to alleviate the autophagy-lysosomal dysfunction commonly observed in AD [56, 57]. In this study, we found that TFEB was decreased in AD, while LAMP1 was increased in AD. The reasons for the discrepancies of the level of TFEB and LAMP1 in AD might be that although the biogenesis of lysosomes is decreased, because of the compromised degradation function of lysosomes, the lysosome become larger and aggregated, thus the Lamp1 is often increased [58].

Furthermore, multimodal intervention not only stimulates lysosomal biogenesis but also preserves the structural integrity of lysosomes. Lysosomal membrane integrity is essential for the degradation of protein aggregates [59, 60]. In the AD mice, permeabilization was increased and lysosomes were enlarged. Multimodal intervention significantly reversed the integrity of lysosomes and decreased the area of each lysosome. The restoration of lysosomal integrity by multimodal intervention in AD mice may be achieved through the modulation of heat shock protein family A (HSPAs). Related studies have shown that the HSPAs may interact with key lysosomal membrane lipids such as bis (monoacylglycerol) phosphate (BMP), thereby preventing permeabilization of the lyosome [35, 36, 61]. Our results showed that in AD model mice, the expression of HSPA1L was significantly decreased, and the integrity of lysosome membrane was compromised. While multimodal intervention significantly increased the expression of HSPA1L and restored the integrity of the lysosomal membrane. Moreover, knockdown of HSPA1L in neuronal cells led to reduced lysosomal membrane integrity, similar to what was observed in AD mice, while upregulation of HSPA1L reversed the disruption of lysosome membrane integrity of AD transgenic mice, thereby reversing the increased accumulation of Aβ and cognitive defects of AD. Therefore, multimodal intervention might restore lysosomal integrity and cognitive defects of AD via increasing the expression level of HSPA1L.

Despite these encouraging findings, several limitations should be acknowledged.

As revealed by our proteomics results, multimodal intervention may exert its beneficial effects through multiple interconnected biological processes. Our research primarily verified the key inflammatory and autophagy–lysosomal pathways. Other important aspects, including synaptic plasticity, neurotrophic support, and metabolic homeostasis, might also play an important role in the beneficial effects of multimodal intervention. Moreover, future research is needed to confirm the neuroprotective effects of multimodal intervention in AD patients. Nevertheless, restoring the function of the autophage-lysosome pathway might be the common pathway through which multimodal intervention exerts its protective effects on cognitive and affective disorders of AD.

## Conclusions

Physical exercise and audiovisual stimulation exert synergistic effects in decreasing inflammation reaction and maintaining the autophagy-lysosomal homeostasis via increasing the biogenesis of lysosomes and restoring the integrity of lysosome membrane, thereby reducing Aβ deposition and tau hyperphosphorylation. These findings underscore the therapeutic potential of multimodal, non-pharmacological strategies for Alzheimer’s disease and provide new insights into comprehensive prevention and treatment approaches. 

## Supplementary Information

Supplementary Material 1.

## Data Availability

The raw data have been uploaded to iProX and are publicly available at https://www.iprox.cn/page/project.html?id=IPX0016828000. All data generated or analyzed in this study are included in this published article and its supplementary information files.

## Abbreviations

AD: Alzheimer's disease
ADE: AD with exercise
ADEVL: AD with multimodal intervention
ADVL: AD with 40 Hz voice and light
APP: Amyloid-beta precursor protein
ATG4B: Autophagy related 4b cysteine peptidase
Aβ: Amyloid β-protein
BMP: Bis(monoacylglycero) phosphate
C1q: Complement component 1q
C1qa: Complement c1q subcomponent subunit A
C1qb: Complement c1q subcomponent subunit B
CD44: CD44 Molecule
DEPs: Differentially expressed proteins
Eif5b: Eukaryotic translation initiation factor 5B
GAPDH: Glyceraldehyde-3-phosphate dehydrogenase
GSEA: Gene set enrichment analysis
HSPA1L: Heat shock protein A1-like
HSPA8: Heat shock protein A8
LAMP1: Lysosomal-associated membrane protein 1
LC3B: Microtubule associated protein 1 light chain 3 beta
LMP: Lysosomal membrane permeabilization
MAPT: Microtubule-associated protein tau
MWM: Morris water maze
NOR: Novel object recognition
p-Tau: Phosphorylated microtubule-associated protein tau
p62: Sequestosome 1
Sacs: Sacsin molecular chaperone
siRNA: Small interfering RNA
STING1: Stimulator of interferon genes protein 1
SYNJ2: Synaptojanin 2
S100a6: S100 calcium binding protein A6
Tau: Microtubule associated protein tau
TEM: Transmission electron microscopy
TFE3: Transcription factor e3
Vcam1: Vascular cell adhesion molecule 1
TFEB: Transcription factor EB
WT: Wild-type
ZKSCAN16: Zinc finger with krab and scan domains 16
